# Systematic Review With Meta-analysis: Safety and Effectiveness of Combining Biologics and Small Molecules in Inflammatory Bowel Disease

**DOI:** 10.1093/crocol/otac002

**Published:** 2022-02-10

**Authors:** Quazim A Alayo, Marc Fenster, Osama Altayar, Kerri L Glassner, Ernesto Llano, Kindra Clark-Snustad, Anish Patel, Lukasz Kwapisz, Andres J Yarur, Benjamin L Cohen, Matthew A Ciorba, Deborah Thomas, Scott D Lee, Edward V Loftus, David I Fudman, Bincy P Abraham, Jean-Frederic Colombel, Parakkal Deepak

**Affiliations:** 1 Department of Internal Medicine, St. Luke’s Hospital, St. Louis, Missouri, USA; 2 Division of Gastroenterology and Inflammatory Bowel Diseases Centre, Washington University in Saint Louis School of Medicine, St. Louis, Missouri, USA; 3 Division of Gastroenterology, Albert Einstein College of Medicine, Montefiore Medical Centre, Bronx, New York, USA; 4 Division of Gastroenterology, Washington University in Saint Louis School of Medicine, St. Louis, Missouri, USA; 5 Division of Gastroenterology and Hepatology, Houston Methodist Hospital, Houston, Texas, USA; 6 Division of Digestive and Liver Diseases, University of Texas Southwestern, Dallas, Texas, USA; 7 Division of Gastroenterology, University of Washington Medical Centre, Seattle, Washington, USA; 8 Division of Gastroenterology, Brooke Army Medical Centre, Fort Sam Houston, Texas, USA; 9 Section of Gastroenterology and Hepatology, Baylor College of Medicine, Houston, Texas, USA; 10 Division of Gastroenterology and Hepatology, Medical College of Wisconsin, Milwaukee, Wisconsin, USA; 11 Department of Gastroenterology, Hepatology, and Nutrition, Digestive Disease and Surgery Institute, Cleveland Clinic, Cleveland, Ohio, USA; 12 Bernard Becker Medical Library, Washington University in Saint Louis, St. Louis, Missouri, USA; 13 Division of Gastroenterology and Hepatology, Mayo Clinic College of Medicine, Rochester, Minnesota, USA; 14 Dr. Henry D. Janowitz Division of Gastroenterology, Icahn School of Medicine at Mount Sinai, New York, New York, USA

**Keywords:** inflammatory bowel diseases, biologics, ulcerative colitis, Crohn’s disease, meta-analysis

## Abstract

**Background:**

Combining biologics and small molecules could potentially overcome the plateau of drug efficacy in inflammatory bowel disease (IBD). We conducted a systematic review and meta-analysis to assess the safety and effectiveness of dual biologic therapy (DBT), or small molecule combined with a biologic therapy (SBT) in IBD patients.

**Methods:**

We searched MEDLINE, EMBASE, Scopus, Web of Science, Cochrane Database of Systematic Reviews, and Clinical trials.gov until November 3, 2020, including studies with 2 or more IBD patients on DBT or SBT. Main outcome was safety assessed as pooled rates of adverse events (AEs) and serious AEs (SAEs) for each combination. Effectiveness was reported as pooled rates of clinical, endoscopic, and/or radiographic response and remission. The certainty of evidence was rated according to the Grades of Recommendation, Assessment, Development, and Evaluation (GRADE) framework.

**Results:**

Of the 3688 publications identified, 13 studies (1 clinical trial, 12 observational studies) involving 266 patients on 7 different combinations were included. Median number of prior biologics ranged from 0 to 4, and median duration of follow-up was 16–68 weeks. Most common DBT and SBT were vedolizumab (VDZ) with anti-tumor necrosis factor (aTNF, *n* = 56) or tofacitinib (Tofa, *n* = 57), respectively. Pooled rates of SAE for these were 9.6% (95% confidence interval [CI], 1.5–21.4) for VDZ-aTNF and 1.0% (95% CI, 0.0–7.6) for Tofa-VDZ. The overall certainty of evidence was very low due to the observational nature of the studies, and very serious imprecision and inconsistency.

**Conclusions:**

DBT or SBT appears to be generally safe and may be effective in IBD patients, but the evidence is very uncertain.

## Lay Summary

Our study suggests that a combination of 2 biologics or a biologic with a small molecule drug appears safe, with no new adverse event signal. These combinations may be potentially effective as a therapeutic option in treatment refractory inflammatory bowel disease patients, but the evidence is very uncertain.

## Introduction

The morbidity associated with persistent inflammation in inflammatory bowel disease (IBD) has spurred a shift in the goals of therapy from the control of symptoms to achieving corticosteroid-free sustained complete remission including endoscopic remission.^1–4^ Since the approval of the first tumor necrosis factor antagonist (anti-TNF) for IBD treatment about 2 decades ago, several other biologics and small molecule drugs (SMDs) including vedolizumab (VDZ), ustekinumab (UST), and tofacitinib (Tofa) have been approved for patients with moderate-to-severe IBD. Despite these new advances, many patients do not achieve sustained complete remission, with about 40% of patients having persistent symptoms and/or mucosal disease after 1 year of biologic therapy and a fifth requiring surgery within 2–5 years of therapy.^5,6^

The failure to achieve sustained complete remission with biologics or SMDs has led to an increasing interest in exploring the therapeutic potential of combining mechanistically different biologics, or biologics with a small molecule therapy in refractory IBD patients. Furthermore, IBD patients often have concomitant extraintestinal manifestations sometimes necessitating combining biologics and/or small molecules. However, there are concerns about the safety of this approach, especially because studies on combination therapies in the rheumatological literature have demonstrated concerning safety profiles.^5^ To date, the only randomized controlled trial (RCT) of combination therapy in IBD compared the combination of infliximab (anti-TNF) with the anti-integrin natalizumab (NAT) to infliximab monotherapy, and it reported no new safety signal.^7^ A prior systematic review and pooled analysis of 7 studies on dual therapy with biologics across a total of 18 patients also reported no new safety signal.^8^ Since this review, several other studies have been published reporting on the safety and effectiveness of dual biologic therapy (DBT) or a biologic plus a small molecule therapy (SBT) in IBD patients.^9–11^ A more recent systematic review and meta-analysis^12^ provided pooled safety and efficacy estimates across all patients on 9 different DBT and SBT that do not account for the significant heterogeneity in individual combination types. It also did not provide point estimates for safety and effectiveness for individual combinations necessary for shared decision making when considering combination therapy in an individual patient.

The aim of this study therefore was to provide a more comprehensive systematic review and meta-analysis to assess the safety and effectiveness of DBTs and SBTs in patients with IBD across the various individual combinations and to identify gaps for future study of this emerging concept in IBD therapy.

## Methods

### Search Strategy and Study Selection

We conducted a systematic review and meta-analysis by systematically searching MEDLINE, EMBASE, Scopus, Web of Science, Cochrane Database of Systematic Reviews, and Clinical trials.gov to identify relevant studies published from the date of each database’s inception up to November 3, 2020, using keywords shown in Supplementary Table S1. The searches were limited to the English language and to human studies. Reference lists of review articles that were published in the same period were also searched. When abstracts were identified, we periodically search for the final publication to be included in the systematic review. We also included studies that some of the authors of this review participated in and were awaiting journals decisions.

Literature search was done by an experienced medical librarian (D.T.). Two authors (Q.A.A. and M.F.) independently screened all article titles and abstracts for relevance, based on inclusion and exclusion criteria. Full texts were retrieved, assessed for eligibility and data extracted independently for each study, using a predefined protocol and recorded on a form using REDCap (Research Electronic Data Capture, version 7.3.5) hosted at Washington University School of Medicine in St. Louis.^13,14^ Any disagreement between investigators was resolved by consensus between them and in discussion with a third author (P.D.). The review was reported in accordance with the MOOSE^15^ and PRISMA^16^ guidelines. The systematic review was registered with PROSPERO, CRD42020183611.

### Inclusion and Exclusion Criteria

Studies were included if they met the following criteria: randomized controlled trials (RCTs) or observational studies; involved 2 or more IBD patients, including IBD-U (IBD-unclassified) patients; patients were treated concurrently with a combination of any 2 of the following biologics (anti-TNFs [infliximab, adalimumab, certolizumab pegol, golimumab], NAT, UST, or VDZ or small molecule therapy [Tofa]); and reported effectiveness and/or safety for the combination. Single case reports and narrative reviews were excluded. Patients on drugs that are not FDA approved for IBD were excluded. In instances of missing or incomplete data, corresponding authors were contacted to obtain additional data.

### Study Outcomes and Data Synthesis

The main outcome of interest was safety assessed as pooled rates of any adverse events (AEs) and serious AEs (SAEs) for each combination. SAEs were defined as AEs which were life-threatening or resulting in a hospitalization, disability, or discontinuation of therapy. Other outcomes included pooled rates of infectious SAEs and gastrointestinal infections. We also assessed effectiveness of combination therapy as pooled rates of clinical response and remission, and endoscopic/radiographic response and remission. Clinical response/remission or endoscopic/radiographic response/remission were taken as reported by the authors regardless of response definition or scoring system used. For sensitivity analysis, we also assessed effectiveness limited to DBT or SBT used primarily for luminal disease. Some of the included studies had patients who underwent multiple therapeutic trials (TTs) and reported effectiveness per trial. However, all included studies reported safety outcomes per patient regardless of number of TTs. We therefore reported safety outcomes per patient and effectiveness per TTs.

### Quality Assessment and Statistical Analysis

We assessed the quality of each study using a methodological quality appraisal tool for case series studies by Moga et al.^17^ We did not use a quantitative score to summarize the risk of bias per study; rather, we based the global judgment on the domains listed above.^18^ Risk of bias of RCTs was assessed using the same tool since the review and meta-analysis will only calculate pooled proportion of a single arm of RCTs (intervention arm) without relative effect size (ES) calculation. Additionally, we also assessed risk of bias in RCTs using the Cochrane Collaboration’s tool for assessing risk of bias in randomized trials.^19^ The overall certainty in evidence was evaluated using the Grading of Recommendations, Assessment, Development, and Evaluation (GRADE) approach.^20^

All statistical analyses were performed using *R* version 4.0.3^21^ and the package *meta* 4.15-1.^22^ The proportions of patients who developed AEs or achieved response or remission were pooled using the Freeman–Tukey double arcsine transformation and the DerSimonian and Laird random-effects model.^23,24^ The random-effects model was selected due to the anticipated clinical heterogeneity.^25^ Where possible, the generalized linear mixed model (GLMM) was used for sensitivity analysis. Heterogeneity between studies was quantified using the *I*-squared (*I*^2^) statistic, with values of <30%, 30%–60%, 61%–75%, and >75% being suggestive of low, moderate, substantial, and considerable heterogeneity, respectively.^26^ Publication bias was assessed using asymmetry tests when appropriate.^27^

### Ethical Considerations

This study is exempt from IRB approval because publicly available data that were part of IRB-approved study protocols was used in data synthesis.

## Results

### Search Result, Study Characteristics, and Quality Assessment

Our search identified 3688 publications. After excluding 775 duplicates and 2884 irrelevant studies following abstract screening, 29 full-text articles were retrieved for further assessment. Of these, 16 were excluded for reasons listed in Supplementary Figure S1, leaving 13 studies eligible for inclusion in the systematic review and meta-analysis.^7,9–11,28–36^ We contacted the authors of 6 studies with insufficient data for additional information.^9–11,31,33,36^

The included 13 studies had a total of 273 patients who underwent 279 TTs. Of these, we excluded 7 patients (8 TTs) who were on a combination including etanercept, apremilast, secukinumab, or ocrelizumab—biologics/SMDs not FDA approved for IBD. A total of 266 patients who underwent 271 TTs of 7 different combination therapies were included in the final systematic review and meta-analysis. There were 188 Crohn’s disease (70.7%), 75 ulcerative colitis (28.2%), and 3 IBD-U (1.1%) patients. Only one of the included studies was a RCT—others were observational studies. Most were single center studies (*n* = 8, 61.5%), carried out in the United States (*n* = 9, 69.2%), and published in 2020 (*n* = 10, 76.9%). The median age of patients in the included studies ranged from 16.9 to 49 years. Fifty-five percent of the patients were female, the median number of prior biologic use across studies ranged from 0 to 4, and the median duration of follow-up ranged from 16 to 68 weeks (Tables 1 and 2). Most patients were started on dual therapy because of active luminal disease. There was significant variation in how clinical and endoscopic effectiveness was assessed across all studies (Table 2). Discontinuation of combination therapy was reported in 72 of 214 TTs (33.6%).

**Table 1.** Baseline information and patient characteristics.

	Study type	Country (single/multicenter)	IBD type, *n* (*N* = 266)^a^	No. of previous biologics, median (IQR/range)	No. of therapeutic trials, *n* (*N* = 271)^a^	Combinatorial treatments	Median duration of follow-up (weeks)	No. of therapeutic trials discontinued DT, *n*/*N* (%)
Sands et al^7^	Randomized controlled trial	United States (multicenter)	CD (52)	0	52	NAT + aTNF	m 32	5/52 (9.6%)
Buer et al^28^	Prospective cohort	Norway (multicenter)	CD (4) UC (6)	NA (R 1–3)	10	VDZ + aTNF	68	2/10 (20.0%)
Mao et al^29^	Case series	United States (single)	CD (3)	3 (IQR 2–5)	3	VDZ + aTNF VDZ + UST	NA	0/3 (0%)
Dolinger et al^32^^b^	Retrospective cohort	United States (single)	CD (7) UC (8) IBD-U (1)	3 (IQR 1–2.5)	16	VDZ + UST Tofa + UST Tofa + VDZ	26	3/16 (18.8%)
Fumery et al^33^	Case series	France (single)	CD (4) UC (1)	2 (IQR 0–4)	5	UST + aTNF VDZ + aTNF	24	NA
Glassner et al^10^	Retrospective cohort	United States (single)	CD (30) UC (18) IBD-U (1)	2 (IQR 1–2)	52	VDZ + aTNF VDZ + UST UST + aTNF Tofa + aTNF Tofa + VDZ	16	NA
Kwapisz et al^9^	Retrospective cohort	United States (single)	CD (14) UC (1)	4 (R 1–7)	15	VDZ + aTNF UST + aTNF VDZ + UST	24	1/15 (6.7%)
Olbjorn et al^34^^b^	Retrospective cohort	Norway (single)	CD (9) UC (4)	1 (R 1–2)	13	VDZ + aTNF UST + aTNF	NA	12/13 (92.3%)
Privitera et al^35^	Retrospective cohort	Italy (multicenter)	CD (10) UC (3)	2.5 (IQR 2–3)	13	VDZ + aTNF UST + aTNF VDZ + UST	28	4/13 (30.8%)
Yang et al^11^	Retrospective cohort	United States/Canada (multicenter)	CD (22)	4 (R 2–5)	24	VDZ + aTNF UST + aTNF VDZ + UST	39	15/24 (62.5%)
Alayo et al^30^	Retrospective cohort	United States (multicenter)	CD (10) UC (25)	2 (IQR 1–3)	35	Tofa + aTNF Tofa + UST Tofa + VDZ	16	20/35 (57.1%)
Lee et al^31^	Retrospective cohort	United States (single)	CD (19)	4 (IQR 3–4)	19	Tofa + aTNF Tofa + UST Tofa + VDZ	42.3	5/19 (26.3%)
Llano et al^36^	Retrospective cohort	United States (single)	CD (3) UC (10) IBD-U (1)	2 (R 1–4)	14	VDZ + aTNF VDZ + UST Tofa + VDZ	31	5/14 (35.7%)

Abbreviations: APR, apremilast; aTNF, anti-tumor necrosis factor; CD, Crohn’s disease; DT, dual therapy; ETA, etanercept; IBD, inflammatory bowel disease; IBD-U, inflammatory bowel disease unclassified; IQR, interquartile range; m, mean; NA, not available; NAT, natalizumab; OCR, ocrelizumab; R, range; SKM, secukinumab; Tofa, tofacitinib; UC, ulcerative colitis; UST, ustekinumab; VDZ, vedolizumab. Patients who were on biologics/small molecule drugs not FDA approved for IBD were not included in this table.

Some patients underwent multiples therapeutic trials.

Study was done in pediatric population.

**Table 2.** Study characteristics—baseline data and definition of outcomes.

	Male (%)	Age (in years)	Baseline CRP/FC^a^	Indication for combination therapy	Clinical effectiveness definitions	Endoscopy effectiveness definitions	EIM assessment
Sands et al^7^	24 (46)	m 39.9 (±12·6)	CRP: m 6.5 mg/L (±11.1)	Active luminal disease	Response: 70-point decrease from baseline in the CDAI score Remission: CDAI <150	NR	NR
Buer et al^28^	5 (50)	^c^(R 22–48)	CRP: 5.45 mg/dL (R 0.6 to 52.4)	Active luminal disease	Remission: HBI ≤4 or pMS ≤1	Response: CD, significant improvement but still with ulceration; UC, decrease in endoscopic Mayo >1 Remission: CD, absence of ulcerations; UC, Mayo <1	NR
Mao et al^29^	3 (75)	NR	CRP: 37.4 mg/L (27.2 to 41.0)	Active luminal disease; extraluminal disease	NR	NR	NR
Dolinger et al^32^^b^	8 (50%)	M 15.9 (IQR 13.5–16.9)	CRP: 5.7 mg/L (1.5 to 23)	Active luminal disease	Remission: CD: wPCDAI ≤12.5; UC/IBD-U: pMS <2	NR	NR
Fumery et al^33^	4 (57.1)	M 49 (IQR 31–53)	NR	Active luminal disease; extraluminal disease; paradoxical adverse event	NR	NR	NR
Glassner et al^10^	16 (32)	m 36.7 (±13·2)	CRP: 5 mg/dL (1.34 to 23.4)	Active luminal disease; extraluminal disease	Remission: HBI < 5, partial Mayo < 3	Remission: SES-CD score 0–2, Rutgeerts score i0–i1, or Mayo score 0	NR
Kwapisz et al^9^	5 (33.3)	M 36	NR	Active luminal disease	Response: CD: PRO UC: partial Mayo score	NR	NR
Olbjorn et al^34^^b^	6 (46)	M 16 (R 11–17.5)	NR	Active luminal disease; extraluminal disease; paradoxical adverse event	NR	NR	Improvement in psoriasis
Privitera et al^35^	7 (44)	M 38 (R 27–69)	NR	Active luminal disease; extraluminal disease	NR	NR	EIM clinical activity was classified as severe, mild, or remission according to clinical judgment
Yang et al^11^	10 (45)	M 35 (IQR 31–43)	CRP: 17 mg/L (11.0 to 24.0)	Active luminal disease	Response: PRO-2 reduction by 8 Remission: PRO-2 <8	Response: >50% reduction in SES-CD or explicitly stated in endo report Remission: SES-CD<3	NR
Alayo et al^30^	17 (49)	M 32 (IQR 26–39)	CRP: 1.35 mg/dL (0.5 to 11.6)	Active luminal disease	Response: >50% reduction in symptoms assessed based on PGA Remission: 100% reduction in symptoms assessed based on PGA	Response: CD: >50% reduction in SES-CD or ≥50% reduction in MaRIAs UC: ≥1 grade reduction in Mayo Remission: CD: SES-CD of 0–2 or Global MaRIAs score of < 6; UC: Mayo subscore of 0 or 1	NR
Lee et al^31^	9 (47)	M 40 (IQR 30–50)	CRP: 3.7 mg/dL (1·7 to14·9)	Active luminal disease; extraluminal disease	Response: ≥3 point decrease in HBI Remission: HBI ≤4	Response: ≥50% decrease in SES-CD Remission: SES-CD ≤3 Healing: SES-CD = 0	NR
Llano et al^36^	7 (50)	M 37 (IQR 28–53)	CRP: 8 mg/L (<5 to 27) FC: 326 mcg/g (130 to >1000)	Active luminal disease	Remission: normalization of HBI/Lichtiger	Response: improvement in endoscopic Mayo	NR

Abbreviations: CD, Crohn’s disease; CDAI, Crohn’s Disease Activity Index; CRP, C-reactive protein; EIM, extraintestinal manifestations; FC, fecal calprotectin; HBI, Harvey–Bradshaw index; IQR, interquartile range; m, mean; MaRIA, Magnetic Resonance Index of Activity; NR, not reported; PGA, Physician Global Assessment; pMS, partial Mayo score; PRO, patient-reported outcome; R, range; SES-CD, Simple Endoscopic Score for Crohn Disease; UC, ulcerative colitis; wPCDAI, weighted Pediatric Crohn’s Disease Activity Index; ±, standard deviation.

Median (interquartile range) except otherwise specified.

Study was done in pediatric population.

Median or mean was not reported.

Eight studies reported outcomes on DBT only,^7,9,11,28,29,33–35^ 3 studies reported outcomes on both DBT and SBT,^10,32,36^ and 2 studies reported outcomes in SBT only.^30,31^ There were 4 DBTs: vedolizumab + ustekinumab (VDZ-UST), vedolizumab + anti-tumor necrosis factor (VDZ-aTNF), ustekinumab + anti-TNF (UST-aTNF) and natalizumab + anti-TNF (NAT-aTNF), and 3 SBTs: tofacitinib + ustekinumab (Tofa-UST), tofacitinib + vedolizumab (Tofa-VDZ), and tofacitinib + anti-TNF (Tofa-aTNF). The most common DBT and SBT were VDZ-aTNF in 56 TTs across 8 studies,^9–11,28,29,34–36^ and Tofa-VDZ in 57 TTs across 5 studies, respectively (Supplementary Table S2).^10,30–32,36^

Overall, the included observational studies had moderate risk of bias (Supplementary Table S3). Most of the studies had adequate ascertainment of exposures and outcomes, as well as reported AEs and duration of follow-up.

### Safety

**Figure 1.**
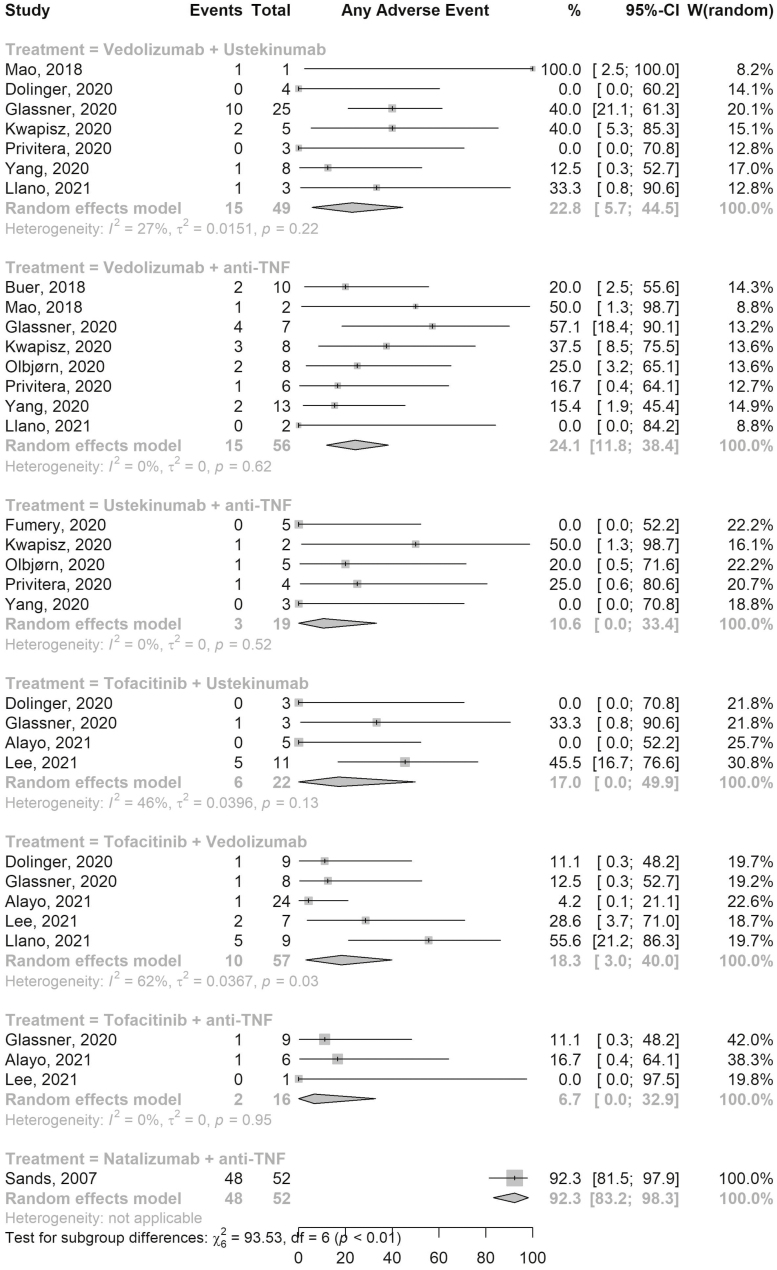
Forest plot of pooled adverse events rates for different combination therapies. Abbreviations: CI, confidence interval; TNF, tumor necrosis factor; W, weights.

AEs were reported for all 7 combination therapies (Figure 1). Fifteen out of 56 patients on VDZ-aTNF developed AEs (pooled AE rate, ES, 24.1%; 95% confidence interval [CI], 11.8–38.4; 8 studies; *I*^2^ 0%) while AEs were reported in 10 of 57 patients on Tofa-VDZ (ES, 18.3%; 95% CI, 3.0–40.0; 5 studies; *I*^2^ 62%). Among the 49 patients on VDZ-UST, 15 patients developed any AE (ES, 22.8%; 95% CI, 5.7–44.5; 7 studies; *I*^2^ 27%), while 2 out of 16 patients on Tofa-aTNF developed AEs (ES, 6.7%; 95% CI, 0.0–32.9; 2 studies; *I*^2^ 0%). The pooled rates of AEs in other combination therapies are shown in Figure 1.

**Figure 2.**
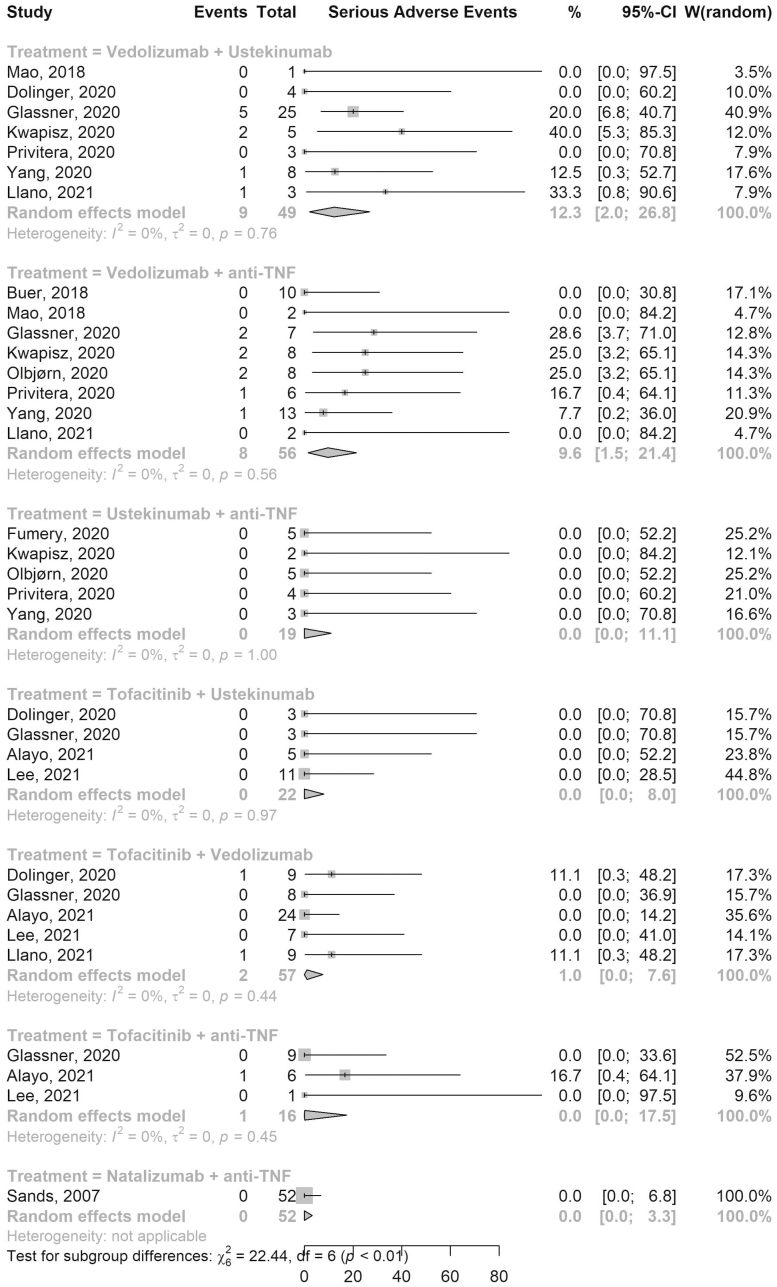
Forest plot of pooled serious adverse events rates for different combination therapies. Abbreviations: CI, confidence interval; TNF, tumor necrosis factor; W, weights.

SAEs were reported by all 13 studies (Figure 2). The pooled rate of SAEs in 56 patients on VDZ-aTNF was 9.6% (95% CI, 1.5–21.4; 8 studies; *I*^2^ 0%) and the rate in 57 patients on Tofa-VDZ was 1.0% (95% CI, 0.0–7.6; 5 studies; *I*^2^ 0%). Among 49 patients on VDZ-UST, the pooled SAE rate was 12.3% (95% CI, 2.0–26.8; 7 studies; *I*^2^ 0%), while pooled SAE rate was 0% in patients on UST-aTNF, Tofa-UST, Tofa-aTNF, and NAT-aTNF (Figure 2). Table 3 shows a pooled list of all AEs reported for each combination.

**Table 3.** List of serious and other adverse events reported by the combination category.

Combinations	Serious adverse events	Other adverse events
Vedolizumab + ustekinumab	Abscesses (abdominal wall, pelvic abscesses, perianal), PICC line infection, sepsis, malnutrition, arthralgia, rotavirus infection/high output ostomy, Acinetobacter bacteremia	*Clostridioides difficile* infection (3), viral enteritis, rotavirus infection, recurrent basal cell skin cancer, sinopulmonary infection (5), viral warts
Vedolizumab + anti-TNF	Peristomal cellulitis, bacterial enteric infection, *Clostridioides difficile* infection, *Salmonella* infection, elevated transaminases, eczema (face, scalp, and body), rash, pneumonia	Bacterial enteric infection, perianal abscess, pneumonia, drug-induced lupus, rash, sinopulmonary infection (4), influenza virus infection, hand–foot–mouth disease
Ustekinumab + anti-TNF	—	Perianal abscess, skin infection, otitis externa, tubo-ovarian abscess
Tofacitinib + ustekinumab	—	Urinary tract infection, sinopulmonary infection, headache, exacerbation of Crohn’s disease, urinary frequency, worsened GERD, rash (2)
Tofacitinib + vedolizumab	Septic arthritis, deep venous thrombosis, *Clostridioides difficile* infection, paresthesia	*Clostridioides difficile* infection (2), pneumonia, high LDL, bacterial enteric infection (*Escherichia coli*),sinopulmonary infection, gluteal abscess, hand injury, basal cell carcinoma, seborrheic dermatitis, rash (2)
Tofacitinib + anti-TNF	*Clostridioides difficile* infection	*Candida* esophagitis, sinopulmonary infection
Natalizumab + anti-TNF	—	Headache (12), fatigue (7), exacerbation of Crohn’s disease (5), dizziness (5), nausea (5), DNA or ANA antibody positive (7), dyspexia (4), abdominal pain (3), arthralgia (3), backpain (3), insomnia (3), pyrexia (3), sinopulmonary infections (8)

Abbreviations: GERD, gastroesophageal reflux disease; LDL, low-density lipoprotein; PICC, peripherally inserted central catheter; TNF, tumor necrosis factor.

Across all combination therapies, the most common SAEs reported were infections, constituting about 75% (15 of 20) of all SAEs. We therefore separately assessed the rate of infectious SAEs (Supplementary Figure S2) and reported the types of infectious SAEs with various combinations (Supplementary Table S4). The pooled rate of infections SAEs in 56 patients on VDZ-aTNF was 3.6% (95% CI, 0.0–13.2; 8 studies; *I*^2^ 0%) with most infections reported being soft tissue infections (*n* = 5, 55.5%). Two infectious SAEs (*Clostridioides difficile* infection and septic arthritis) were reported in 57 patients on Tofa-VDZ with a pooled rate of 1.0% (95% CI, 0.0–7.6; 5 studies; *I*^2^ 0%). A pooled infectious SAE rate of 7.7% (95% CI, 0.2–20.9; 7 studies; *I*^2^ 0%) was reported among the 49 patients on VDZ-UST. Similar to the overall SAE rates, the pooled infections SAE rate was 0% in patients on UST-aTNF, Tofa-UST, Tofa-aTNF, and NAT-aTNF. The pooled rates of gastrointestinal infections in all combination therapies are shown in Supplementary Figure S3. None of the studies assessing SBT reported a case of herpes zoster (HZ) reactivation. One study reported a recurrent basal cell skin cancer in a patient on VDZ-UST.^11^ The patient was reported to have recurrent history of this cancer prior to initiation of DBT.

### Effectiveness

**Figure 3.**
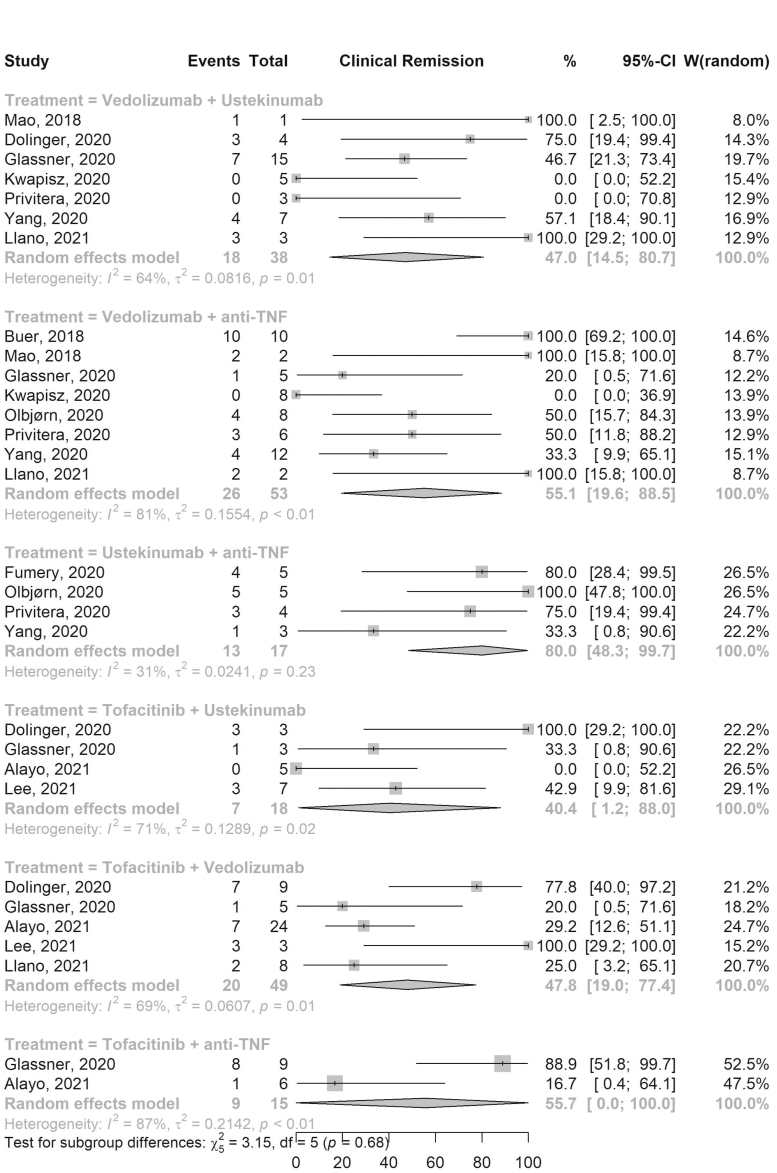
Forest plot of pooled clinical remission rates for different combination therapies. Abbreviations: CI, confidence interval; TNF, tumor necrosis factor; W, weights.

Clinical response was assessed in all 13 studies and clinical remission was reported in all but 1 study.^7^ The pooled clinical response (Supplementary Figure S4) and remission (Figure 3) rates among patients on VDZ-aTNF were 77.9% (95% CI, 51.3–97.2; 8 studies; 53 TTs; *I*^2^ 66%) and 55.1% (95% CI, 19.6–88.5; 8 studies; 53 TTs; *I*^2^ 81%), respectively. Among patients on Tofa-VDZ the pooled clinical response and remission rates were 59.9% (95% CI, 37.2–80.8; 5 studies; 49 TTs; *I*^2^ 43%) and 47.8% (95% CI, 19.0–77.4; 5 studies; 49 TTs; *I*^2^ 69%), respectively. VDZ-UST had a pooled clinical response and remission rates of 83.9% (95% CI, 66.4–96.8; 7 studies; 38 TTs; *I*^2^ 0%) and 47.0% (95% CI, 14.5–80.7; 7 studies; 38 TTs; *I*^2^ 64%), respectively. The pooled clinical response and remission rates for other combination therapies are shown in Supplementary Figure S4 and Figure 3, respectively. Similar rates were observed when analysis was limited to patients who were on dual therapy primarily due to active luminal disease only (Supplementary Figures S5 and S6) and when GLMM was used (Supplementary Figures S7 and S8).

Nine studies reported endoscopic and/or radiologic outcomes after initiation of combination therapy,^9–11,28–31,33,36^ with 6 of these studies providing the criteria for endoscopic assessments.^10,11,28,30,31,36^ We reported the pooled endoscopic/radiologic response/remission rates for all combinations except NAT-aTNF (Supplementary Figures S9 and S10). The pooled endoscopic/radiologic response and remission rates among patients on VDZ-aTNF were 38.2% (95% CI, 19.5–58.4; 5 studies; 35 TTs; *I*^2^ 0%) and 18.0% (95% CI, 1.6–41.8; 5 studies; 35 TTs; *I*^2^ 32%), respectively. The corresponding rates among patients on Tofa-VDZ were 46.2% (95% CI, 20.4–73.0; 4 studies; 31 TTs; *I*^2^ 40%) and 24.6% (95% CI, 6.4–47.6; 4 studies; 31 TTs; *I*^2^ 25%). Pooled endoscopic/radiologic effectiveness rates observed when analysis was limited to patients who were on dual therapy primarily due to active luminal disease and when GLMM was used are shown in Supplementary Figures S11–S14.

Based on the GRADE framework, the certainty in evidence was very low for all outcomes because of the observational nature of the studies, very serious imprecision (due to the small number of events and patients), and very serious inconsistency between the studies. Due to the small number of studies and substantial heterogeneity, we could not assess for publication bias. There is empirical evidence of publication bias favoring positive results when it comes to case reports and case series in general.^37^

## Discussion

Here, we present a systematic review and meta-analysis pooling data from 13 studies across different biologics and small molecules to assess the safety and effectiveness of DBT and SBT in IBD patients. We demonstrated that, based on current available data, DBT and SBT appear to be safe with a pooled SAEs rates ranging from 0% (for UST-aTNF [95% CI, 0.0–11.1], Tofa-UST [95% CI, 0.0–8.0], Tofa-aTNF [95% CI, 0–17.5], and NAT-aTNF [95% CI, 0–3.3]) to 12.3% (VDZ-UST [95% CI, 2.0–26.8]). None of the combination therapies assessed in this review revealed any new safety signal. We also observed that DBT and SBT were effective for refractory IBD with pooled clinical remission rates ranging from 40.4% (for Tofa-UST [95% CI, 1.2–88.0]) to 80.0% (UST-aTNF [95% CI, 48.3–99.7]) and endoscopic/radiologic remission rates ranging from 18.0% (for VDZ-aTNF [95% CI, 1.6–41.8]) to 37.4% (Tofa-UST [95% CI, 9.5–69.4]).

Previous reviews summarizing studies on safety and efficacy of combination biologics and/or small molecules are either narrative reviews,^5,34^ including mostly case reports with small sample size,^5,8^ or had no patients on SBT.^5,8,34^ A more recent systematic review and meta-analysis by Ahmed et al^12^ reported a single pooled safety and effectiveness estimate for 279 patients on 9 different dual biologics and/or small molecules therapies without providing estimates for each individual combination therapy. These estimates therefore do not account for the significant heterogeneity in combination types. Furthermore, Ahmed et al also included patients on biologics or small molecules which are not currently approved for IBD treatments, therefore diluting the estimates. Therefore, our study is a more comprehensive updated systematic review and meta-analysis with a large pool of patients providing pooled safety and effectiveness estimates for individual DBT or SBT in patients with refractory IBD.

The increasing prevalence of IBD is associated with major healthcare and economic burden,^38^ and the chronic inflammatory nature of IBD results in long-term complications, frequent hospitalizations, need for multiple surgeries and decreased quality of life.^39^ Although the use of biologics and SMDs has significantly improved outcomes in IBD, significant therapeutic gaps still exist. There has been an increasing interest in exploring the therapeutic potential of combining mechanistically different biologics and/or SMDs to further bridge the remaining gap in therapy. However, there are concerns about the safety of this approach.

These safety concerns come largely from experience in using combination biologic therapy in rheumatological diseases, for which more high-quality prospective studies have been performed. A meta-analysis by Boleto et al which included a total of 623 RA patients (410 on DBT and 213 on single biologic) with a median follow-up of 9.5 months, suggested that DBT in RA appeared to increase the risk of SAEs (14.9% vs 6.0%).^40^ However, the biologics used in these studies, such as abatacept, rituximab, or tocilizumab are not used in IBD, and generally tend to have poorer safety profiles compared to the biologics/SMD reported in our meta-analysis. The safety profile of gut-specific anti-integrin agents such as VDZ also provides some reassurance. In addition, the only RCT published on DBT in IBD, which compared a combination of infliximab (an aTNF) and NAT (anti-integrin agent) to infliximab alone in refractory CD patients, reported favorable safety outcomes.^7^ Furthermore, individual retrospective case series and previous pooled analyses of these reports have all reported no new safety concerns.^8^ Our study adds to this body of knowledge highlighting that taken together, the preponderance of evidence on DBT and SBT in IBD suggest they are safe.

The safety profiles of the combinations we reported in this meta-analysis are similar to what have been reported for individual biologics or SMDs when used as monotherapy in IBD. In the VARSITY trial, a head-to-head RCT comparing VDZ to adalimumab in moderate-to-severe UC patients, the SAE rates in the VDZ and adalimumab groups were 13.7% and 11.0%, respectively.^41^ Similar SAE rates were reported in a real-world multicenter study comparing the safety profile of VDZ to aTNFs (14% vs 14%).^42^ For UST, Sandborn et. al. reported SAE rate of 4.4% (95% CI, 3.5–5.6) and infectious SAE rate of 5.0 (95% CI, 4.0–6.2) in the UST group in a pooled safety analysis of results of phase 2/3 trials of UST in IBD,^43^ and a similar SAEs rate (5.5%) was reported in a pooled analysis of real-world data on UST for Crohn’s disease.^44^ The overall and infectious SAE rates reported in these studies fall within the range of SAE rates we found for DBT in our study (range, overall, 0%–12.3%; infectious, 0%–7.7%). Similarly, the SAE rate for SBT in our meta-analysis (range, 0%–1.0%) is numerically lower than the 5.8% we recently reported in the real-world tofacitinib monotherapy study (TROPIC) as well as the 3.6% and 5.1% reported in the Tofa arm of the pivotal induction trial and maintenance trials, respectively.^45,46^ Unlike the Tofa monotherapy studies that have shown a dose-dependent increase in the risk of HZ infection,^47^ none of the studies assessing SBT reported a case of HZ infection, although this may be due to the relatively short duration of follow-up in these studies or small sample size.

In addition to the favorable safety profile, our results also suggest that combination therapy may be effective. However, this effectiveness should be interpreted with caution given the overall very low certainty with substantial heterogeneity in how and when the response was assessed across all studies, as well as the less stringent definition of response in real-world data compared to RCTs. Despite this, the clinical and endoscopic response and remission rates reported in this meta-analysis are similar to those reported for biologic monotherapies in other real-world studies of UST and VDZ despite a patient population in our analyses that is more refractory to medical therapy overall.^44,48,49^ However, most of the studies in our analysis have a short duration of follow-up, and long-term durability of these clinical and endoscopic outcomes is unclear. Future studies are therefore needed to assess if the clinical and endoscopic improvement rates reported in this meta-analysis persist in RCTs and real-world studies with longer duration of follow-up.

Our systematic review with meta-analysis has several strengths. We utilized a comprehensive search strategy using multiple databases. Secondly, it represents the most comprehensive meta-analysis with a large of patients to assess the safety and effectiveness on individual DBTs and SBTs in refractory IBD patients. Our study also provides important estimates on the AEs and treatment response rates for different combination therapies that can guide shared decision making for clinicians and patients considering DBT or SBT in the setting of refractory IBD and to guide the design of future prospective studies addressing similar questions. Finally, we have also included data from pediatric studies allowing pediatric gastroenterologists to use the information presented here in their decision making.

However, there are a few limitations. Since most of the studies included in this meta-analysis are observational, this analysis is prone to the bias inherent in the included studies. Indeed, we observed substantial degree of heterogeneity between studies, which is likely due to differences in selection of study population, small sample size, difference in outcome definition, and potential selective reporting of positive outcomes in the original studies. These factors are also responsible for the overall very low certainty in the results presented here and our inability to examine effectiveness by IBD subtype. Furthermore, because of the small sample size within each combination therapy, and the significant heterogeneity between the studies, we were unable to do a head-to-head comparison of the different DBTs or SBTs. Lastly, most of the included studies have a short duration of follow-up and definitions of AEs different from clinical trials. This may partly explain the relatively low rates of AEs, and the high effectiveness rates, however the rates of SAEs are well defined and reliable.

Despite these limitations, in the absence of rigorously designed prospective studies with long-term follow-up data, our results provide the most updated evidence yet on the safety and effectiveness of DBT and SBT across a large group of IBD patients. This information can guide gastroenterologists in making important treatment decisions as they care for patients with refractory luminal or extraintestinal manifestations of IBD. While the absence of new safety signals in this study is reassuring, the overall result of this meta-analysis should be interpreted with caution given the short duration of follow-up and very low certainty of evidence. It is prudent that clinicians adequately weigh the potential risks and benefits and cost-effectiveness of combining biologics and/or small molecules until more rigorous RCTs data are available to affirm these findings.

## Conclusion

In conclusion, this systematic review and meta-analysis demonstrated that a combination of 2 biologics or a biologic with a SMD may be safe and potentially effective in patients with refractory IBD. We did not observe any new safety signal across all combinations. However, these findings need to be confirmed in prospective studies. The results of the on-going EXPLORER trial, an open-label study evaluating the combination of VDZ, adalimumab, and methotrexate (ClinicalTrials.gov Identifier: NCT02764762), will likely provide more precise estimates of safety and effectiveness of this triple combination.

## Supplementary Material

otac002_suppl_Supplementary_Material

## Data Availability

All data are publicly available in the respective publication of included studies. We have also included other data we obtained directly from authors of included studies in our result sections as well as in the supplementary data.
