# Supplementary material for: Systematic Review With Meta-analysis: Safety and Effectiveness of Combining Biologics and Small Molecules in Inflammatory Bowel Disease
Source: Crohns Colitis 360. 2022 Feb 10;4(1):otac002. doi: 10.1093/crocol/otac002 (PMC8924906; doi:10.1093/crocol/otac002)
Supplement: otac002_suppl_Supplementary_Material [file otac002_suppl_supplementary_material.docx]

**Supplementary Data**

**Supplementary Table 1: Key words for database search**

| **Database** | **Search Keywords** |
| --- | --- |
| **MEDLINE** | Exp Inflammatory Bowel Diseases OR exp crohn disease OR exp colitis, ulcerative OR inflammatory bowel disease* OR crohn* disease OR “ulcerative colitis”  AND  Exp Drug therapy combination OR combination biologic* therap* OR combination biologic* treatment OR dual biologic* therap* OR dual biological therapy [tiab] OR concurrent treatment OR combination therapy OR simultaneous treatment OR co-treatment OR double biologic* therapy OR “concomitant treatment” OR combination biological agents[tiab]    AND  Exp Antibodies, Monoclonal, Humanized OR exp Tumour necrosis factor-alpha OR “anti-TNF-a” OR “anti-tumour necrosis factor*” OR immunomodulators OR Infliximab [tiab] OR Certolizumab[tiab] OR Golimumab [tiab] OR Adalimumab [tiab] OR Natalizumab[tiab] OR Tofacitinib [tiab] OR Filgotinib [tiab] OR Upadacitinib [tiab] OR tocilizumab [tiab] OR Etanercept [tiab] OR Anakinra [tiab] OR Abatacept [tiab] OR Rituximab[tiab] OR Tildrakizumab [tiab] OR Risankizumab [tiab] OR guselkumab[tiab] OR Ozanimod [tiab] OR Fingolimod [tiab] OR Etrolizumab [tiab] OR Vedolizumab [tiab] OR Natalizumab [tiab] OR Etrasimod [tiab] OR Amiselimod [tiab] OR Ustekinumab[tiab] Infliximab OR Certolizumab OR Golimumab OR Adalimumab OR Natalizumab OR Tofacitinib OR Filgotinib OR Upadacitinib OR tocilizumab OR Etanercept OR Anakinra OR Abatacept OR Rituximab OR Tildrakizumab OR Risankizumab OR Guselkumab OR Ozanimod OR Fingolimod OR Etrolizumab OR Vedolizumab OR Natalizumab OR Etrasimod OR Amiselimod OR Ustekinumab |
| **EMBASE** | (‘Crohn disease’/exp OR ‘colon crohn disease’/exp OR ‘colitis’/exp OR ‘inflammatory bowel disease’/exp/mj OR ‘ulcerative colitis’/exp OR colitis:ti,ab OR ((crohn* NEAR/1 disease):ti,ab OR (inflammatory NEAR/1 bowel NEAR/1 disease*)):ti,ab)  AND  ('combination drug therapy'/exp OR ((combination NEAR/1 biologic* NEAR/1 therap*):ti,ab OR (dual NEAR/1 biolog* NEAR/1 therap*):ti,ab OR (concurrent NEAR/1 treatment):ti,ab OR (simultaneous NEAR/1 treatment):ti,ab OR (co-treatment):ti,ab OR (double NEAR/1 biological NEAR/1 therapy ):ti,ab OR (combination NEAR/1 biologic* NEAR/1 treatment*)):ti,ab)  AND  (‘immunosuppressive agent’/exp OR ‘tumour necrosis factor inhibitory’/exp OR ‘anti-TNF-a’:ti,ab OR Infliximab:ti,ab OR Certolizumab:ti,ab or Golimumab:ti,ab OR Adalimumab:ti,ab OR Natalizumab:ti,ab OR Tofacitinib:ti,ab OR Filgotinib:ti,ab OR Upadacitinib:ti,ab OR tocilizumab:ti,ab OR etanercept:ti,ab OR anakinra:ti,ab OR abatacept:ti,ab OR rituximab:ti,ab OR Tildrakizumab:ti,ab OR Risankizumab:ti,ab OR guselkumab:ti,ab OR Ozanimod:ti,ab) OR Fingolimod:ti,ab OR Etrolizumab:ti,ab OR Vedolizumab:ti,ab OR Natalizumab:ti,ab OR Etrasimod:ti,ab OR Amiselimod:ti,ab OR (immunosuppressive NEAR/1 agent*):ti,ab) |
| **Web of Science core collection** | TS=((crohn* NEAR/1 disease) OR (colitis) OR (inflammatory NEAR/1 bowel NEAR/1 disease) OR (ulcerative NEAR/1 colitis) OR (refractory NEAR Crohn* NEAR/1 disease))  AND  TI=((drug NEAR/1 therapy NEAR/1 combination) OR (combination NEAR/1 biologic* therap*) OR (combination NEAR/1 biologic* NEAR/1 treatment) OR (dual NEAR/1 biologic* NEAR/1 therap*) OR (concurrent NEAR/1 treatment) OR (simultaneous NEAR/1 treatment) OR (combination NEAR/1 agents) OR (co-treatment) OR (double NEAR/1 biologic* NEAR/1 therap*) OR (combination NEAR/1 biological NEAR/1 agents) OR (combination NEAR/1 biologic))  AND  TS= (Antibodies NEAR/1 Monoclonal NEAR/1 Humanized) OR (immunomodulators) OR Infliximab OR Certolizumab OR Golimumab OR Adalimumab OR Natalizumab OR Tofacitinib OR Filgotinib OR Upadacitinib OR tocilizumab OR Etanercept OR Anakinra OR Abatacept OR Rituximab OR Tildrakizumab OR Risankizumab OR guselkumab OR Ozanimod OR Fingolimod OR Etrolizumab OR Vedolizumab OR Natalizumab OR Etrasimod OR Amiselimod OR Ustekinumab |
| **Scopus** | ( TITLE-ABS-KEY ( "Crohn disease" ) OR TITLE-ABS-KEY ( crohn W/1 disease ) OR TITLE-ABS-KEY ( colitis ) OR TITLE-ABS-KEY ( "inflammatory bowel disease" ) OR TITLE-ABS-KEY ( inflammatory W/1 bowel W/1 disease ) OR TITLE-ABS-KEY ( "ulcerative colitis" ) OR TITLE-ABS-KEY ( ulcerative W/1 colitis )  AND  ( TITLE-ABS-KEY ( "tumour necrosis factor inhibitor" ) OR TITLE-ABS-KEY ( tumour W/1 necrosis W/1 factor W/1 inhibitor ) OR TITLE-ABS-KEY ( "antibodies monoclonal humanized" ) OR TITLE-ABS-KEY ( antibodies W/1 monoclonal W/1 humanized ) OR TITLE-ABS-KEY ( "immunosuppressive agent" ) OR TITLE-ABS-KEY ( immunosuppressive W/1 agent* ) OR TITLE-ABS-KEY ( anti-tnf-a ) OR TITLE-ABS-KEY ( infliximab ) OR TITLE-ABS-KEY ( certolizumab ) OR TITLE-ABS-KEY ( golimumab ) OR TITLE-ABS-KEY ( adalimumab ) OR TITLE-ABS-KEY ( natalizumab ) OR TITLE-ABS-KEY ( tofacitinib ) OR TITLE-ABS-KEY ( filgotinib ) OR TITLE-ABS-KEY ( upadacitinib ) OR TITLE-ABS-KEY ( tocilizumab ) OR TITLE-ABS-KEY ( etanercept ) OR TITLE-ABS-KEY ( anakinra ) OR TITLE-ABS-KEY ( abatacept ) OR TITLE-ABS-KEY ( rituximab ) OR TITLE-ABS-KEY ( tildrakizumab ) OR TITLE-ABS-KEY ( risankizumab ) OR TITLE-ABS-KEY ( guselkumab ) OR TITLE-ABS-KEY ( ozanimod ) OR TITLE-ABS-KEY ( fingolimod ) OR TITLE-ABS-KEY ( etrolizumab ) OR TITLE-ABS-KEY ( vedolizumab ) OR TITLE-ABS-KEY ( natalizumab ) OR TITLE-ABS-KEY ( etrasimod ) OR TITLE-ABS-KEY ( amiselimod ) OR TITLE-ABS-KEY (Ustekinumab)  AND  ( TITLE-ABS-KEY ( "drug therapy combination" ) OR TITLE-ABS-KEY ( drug* W/1 therapy W/1 combination ) OR TITLE-ABS-KEY (“concurrent treatment”) OR TITLE-ABS-KEY (“simultaneous treatment”) OR TITLE-ABS-KEY (“double biological therapy”) OR TITLE-ABS-KEY ( "dual biological therapy" ) OR TITLE-ABS-KEY ( dual W/1 biological W/1 therap* ) OR TITLE-ABS-KEY ( combination W/1 biologic* W/1 therap* ) OR TITLE-ABS-KEY ( combination W/1 biologic* W/1 treatment ) ) |
| **Wiley's Cochrane Database of Systematic Reviews** | "inflammatory bowel disease” OR “crohn* disease” OR colitis  AND  “dual biological therapy” OR “combination drug therapy” OR “concurrent treatment” OR “simultaneous treatment” OR co-treatment  AND  Infliximab OR Certolizumab OR Golimumab OR Adalimumab OR Natalizumab OR Tofacitinib OR Filgotinib OR Upadacitinib OR tocilizumab OR Etanercept OR Anakinra OR Abatacept OR Rituximab OR Tildrakizumab OR Risankizumab OR guselkumab OR Ozanimod OR Fingolimod OR Etrolizumab OR Vedolizumab OR Natalizumab OR Etrasimod OR Amiselimod OR Ustekinumab |
| **Clinical trials.gov** | Inflammatory bowel disease OR crohn* disease OR colitis  AND  dual combination therapy OR combination drug therapy OR co-treatment OR simultaneous treatment  AND  Infliximab OR Certolizumab OR Golimumab OR Adalimumab OR Natalizumab OR Tofacitinib OR Filgotinib OR Upadacitinib OR tocilizumab OR Etanercept OR Anakinra OR Abatacept OR Rituximab OR Tildrakizumab OR Risankizumab OR guselkumab OR Ozanimod OR Fingolimod OR Etrolizumab OR Vedolizumab OR Natalizumab OR Etrasimod OR Amiselimod OR Ustekinumab |

**Supplementary Table 2: Different Combination Therapies Reported in the Systematic Review and Meta-Analysis**

| **Combination therapy** | **Number of studies** | **Therapeutic trials, N** | **IBD type (N)** | **Type of Anti-TNF (N)** | **Reference** |
| --- | --- | --- | --- | --- | --- |
| **Vedolizumab + Ustekinumab** | 7 | 49 | CD (44) UC (4) IBD-U (1) | - | (1-7) |
| **Vedolizumab + anti-TNF** | 8 | 56 | CD (40) UC (15) IBD-U (1) | Infliximab (26), Adalimumab (15), Golimumab (8), Certolizumab pegol (7) | (1, 3-9) |
| **Ustekinumab + anti-TNF** | 5 | 19 | CD (18) UC (1) | Infliximab (10), Adalimumab (5), Golimumab (3), Certolizumab pegol (1) | (4, 6, 7, 9, 10) |
| **Tofacitinib + Ustekinumab** | 4 | 22 | CD (19) UC (2)  IBD-U (1) | - | (2, 3, 11, 12) |
| **Tofacitinib + Vedolizumab** | 5 | 57 | CD (16) UC (41) | - | (2, 3, 5, 11, 12) |
| **Tofacitinib + anti-TNF** | 3 | 16 | CD (4) UC (12) | Infliximab (10),  Golimumab (4), Certolizumab pegol (2) | (3, 11, 12) |
| **Natalizumab + anti-TNF** | 1 | 52 | CD (52) | Infliximab (52) | (13) |

CD, Crohn’s disease; IBD-U, Inflammatory bowel disease- unclassified; TNF, Tumour Necrosis Factor, UC, Ulcerative colitis.

**Supplementary Table 3: Risk of Bias in Included Observational Studies Assessed Using the 18-criteria Quality Appraisal Tool for Case series by Moga et al.^14^**

| **Study** | **Study Objective** | **Study population** | | | | | **Intervention and co-intervention** | | **Outcome measure** | | | **Statistical analysis** | **Results and conclusion** | | | | | **Competing interest and source of support** |
| --- | --- | --- | --- | --- | --- | --- | --- | --- | --- | --- | --- | --- | --- | --- | --- | --- | --- | --- |
|  | **1** | **2** | **3** | **4** | **5** | **6** | **7** | **8** | **9** | **10** | **11** | **12** | **13** | **14** | **15** | **16** | **17** | **18** |
| Sands et al  (2007) ^13^* | ✓ | ✓ | ✓ | ✓ | ✓ | ✓ | ✓ | ✓ | ✓ | ✓ | ✓ | ✓ | ✓ | ✓ | ✕ | ✓ | ✓ | ✓ |
| Buer et al  (2018) ^8^ | ✓ | ✓ | ✕ | ✓ | ✓ | ✕ | ✓ | ✕ | ✓ | ✓ | ✕ | ✓ | ✓ | ✓ | ✕ | ✓ | ✓ | ✓ |
| Mao et al  (2018) ^1^ | ✓ | ✓ | ✕ | ✓ | ✕ | ✓ | ✓ | ✕ | ✓ | ✓ | ✕ | ✓ | ✓ | ✓ | ✕ | ✓ | ✓ | ✓ |
| Dolinger et al  (2020)† ^2^ | ✓ | ✓ | ✕ | ✓ | ✕ | ✓ | ✓ | ✓ | ✓ | ✓ | ✓ | ✓ | ✓ | ✓ | ✓ | ✓ | ✓ | ✓ |
| Fumery et al  (2020) ^10^ | ✓ | ✓ | ✕ | ✕ | ✕ | ✕ | ✓ | ✕ | ✕ | ✓ | ✓ | ✕ | ✓ | ✓ | ✕ | ✓ | ✓ | ✓ |
| Glassner et al  (2020) ^3^ | ✓ | ✓ | ✕ | ✓ | ✕ | ✕ | ✓ | ✕ | ✓ | ✓ | ✓ | ✓ | ✓ | ✕ | ✓ | ✓ | ✓ | ✓ |
| Kwapisz et al  (2020) ^4^ | ✓ | ✓ | ✕ | ✓ | ✕ | ✕ | ✓ | ✕ | ✕ | ✓ | ✓ | ✓ | ✓ | ✓ | ✕ | ✓ | ✓ | ✓ |
| Olbjorn et al  (2020)† ^9^ | ✓ | ✓ | ✕ | ✓ | ✕ | ✓ | ✓ | ✓ | ✕ | ✕ | ✓ | ✕ | ✕ | ✕ | ✕ | ✓ | ✓ | ✓ |
| Privitera et al  (2020) ^6^ | ✓ | ✓ | ✓ | ✕ | ✕ | ✕ | ✓ | ✓ | ✕ | ✓ | ✓ | ✕ | ✓ | ✓ | ✕ | ✓ | ✓ | ✓ |
| Yang et al  (2020) ^7^ | ✓ | ✓ | ✓ | ✓ | ✕ | ✕ | ✓ | ✓ | ✓ | ✓ | ✓ | ✓ | ✓ | ✓ | ✓ | ✓ | ✓ | ✓ |
| Alayo et al  (2021) ^11^ | ✓ | ✓ | ✕ | ✕ | ✓ | ✓ | ✓ | ✕ | ✕ | ✕ | ✕ | ✕ | ✓ | ✓ | ✕ | ✓ | ✓ | ✓ |
| Lee et al (2021) ^12^ | ✓ | ✓ | ✓ | ✓ | ✕ | ✕ | ✓ | ✓ | ✓ | ✓ | ✓ | ✓ | ✓ | ✓ | ✓ | ✓ | ✓ | ✓ |
| Llano et al  (2021) ^5^ | ✓ | ✓ | ✕ | ✓ | ✕ | ✕ | ✓ | ✓ | ✕ | ✓ | ✓ | ✕ | ✓ | ✓ | ✓ | ✓ | ✓ | ✓ |

**Key:** ✓ **criterion met; x criterion not met; Study objective** 1. Is the hypothesis/aim/objective of the study clearly stated in the abstract, introduction or methods section? **Study population** 2. Are the characteristics of the participants included in the study described? 3. Were the cases collected in more than one centre? 4. Are the eligibility criteria (inclusion and exclusion criteria) to enter the study explicit and appropriate? 5. Were participants recruited consecutively? 6. Did participants enter the study at a similar point in the disease? **Intervention and co-intervention** 7. Was the intervention clearly described in the study? 8. Were additional interventions (co-interventions) clearly reported in the study? **Outcome measures** 9. Are the outcome measures clearly defined in the introduction or methods section? 10. Were relevant outcomes appropriately measured with objective and/or subjective methods? 11. Were outcomes measured before and after intervention? **Statistical analysis** 12. Were the statistical tests used to assess the relevant outcomes appropriate? **Results and conclusions** 13. Was the length of follow-up reported? 14. Was the loss to follow-up reported? 15. Does the study provide estimates of the random variability in the data analysis of relevant outcomes? 16. Are adverse events reported? 17. Are the conclusions of the study supported by results? **Competing interest and source of support** 18. Are both competing interest and source of support for the study reported? †, Study was done in paediatric population; *, only the natalizumab and infliximab arm of the randomized controlled trial was assessed.

**Supplementary Figure 1: Flow Chart outlining the study screening and selection process**

**

**Supplementary Figure 2: Forest plot of pooled infectious serious adverse events rates for different combination therapies**

**
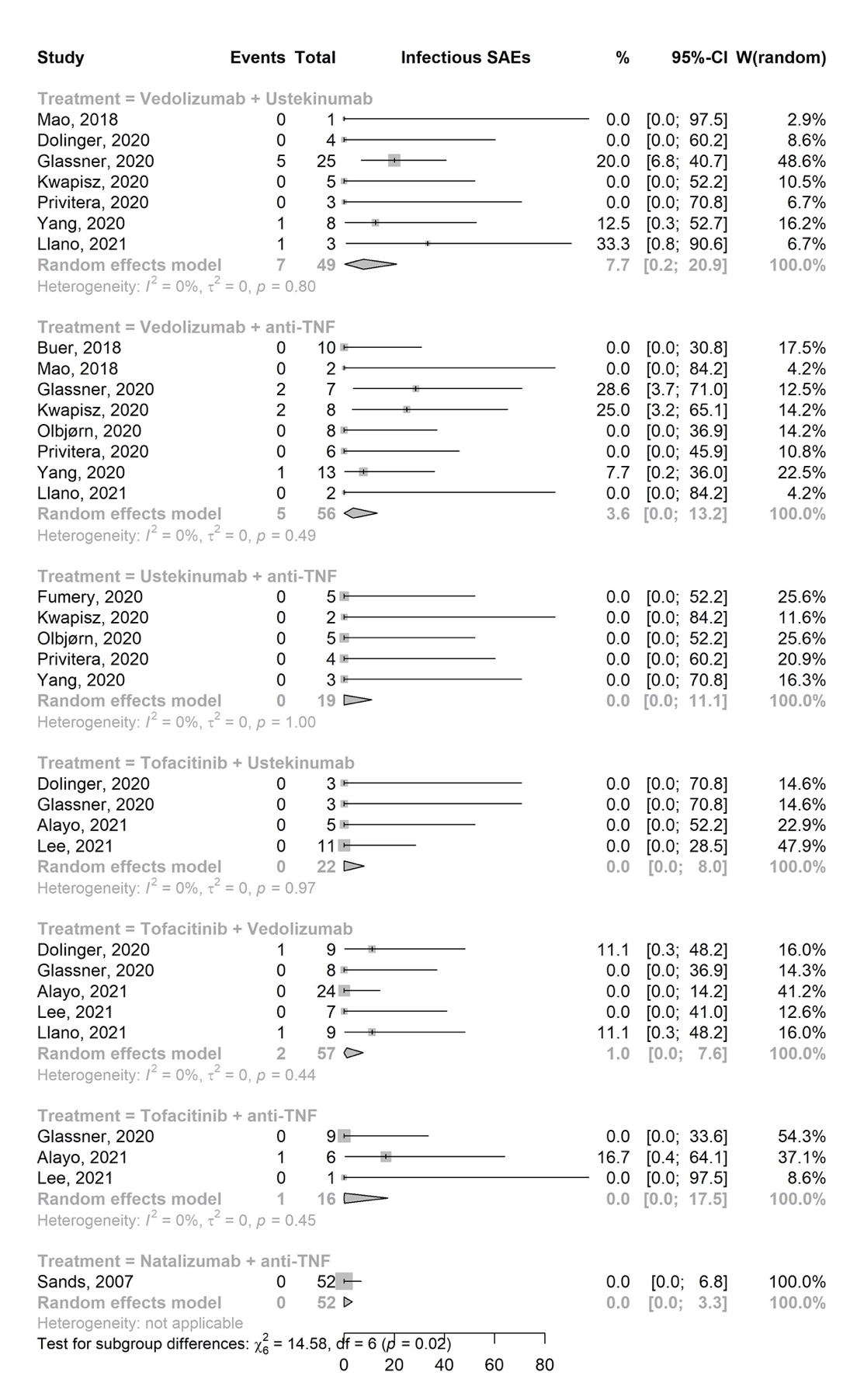
**

CI, Confidence interval; TNF, Tumour necrosis factor; W, Weights.

**Supplementary Table 4: Types of Infectious Serious Adverse Events by the Combination Category**

| **Combination therapy** | **Gastrointestinal** | **Sinopulmonary** | **Soft tissue** | **Joints** | **Others** |
| --- | --- | --- | --- | --- | --- |
| Vedolizumab + Ustekinumab | 1 (Rotavirus infection) | - | 5 (Abscesses (abdominal wall, pelvic, peri-anal) | - | 3 (PICC line infection, sepsis, Acinetobacter bacteraemia) |
| Vedolizumab + anti-TNF | 3 (bacterial enteric infection, *Clostridioides difficile* infection, salmonella infection) | 1 (Pneumonia) | 1 (Peristomal cellulitis) | - | - |
| Ustekinumab + anti-TNF | - | - | - | - | - |
| Tofacitinib + Ustekinumab | - | - | - | - | - |
| Tofacitinib + Vedolizumab | 1 (*Clostridioides difficile* infection) | - | - | 1 (Septic arthritis) | - |
| Tofacitinib + anti-TNF | 1 (*Clostridioides difficile* infection) | - | - | - | - |
| Natalizumab + anti-TNF | - | - | - | - | - |

PICC, Peripherally inserted central catheter; TNF, Tumour Necrosis Factor

**Supplementary Figure 3: Gastrointestinal infections**


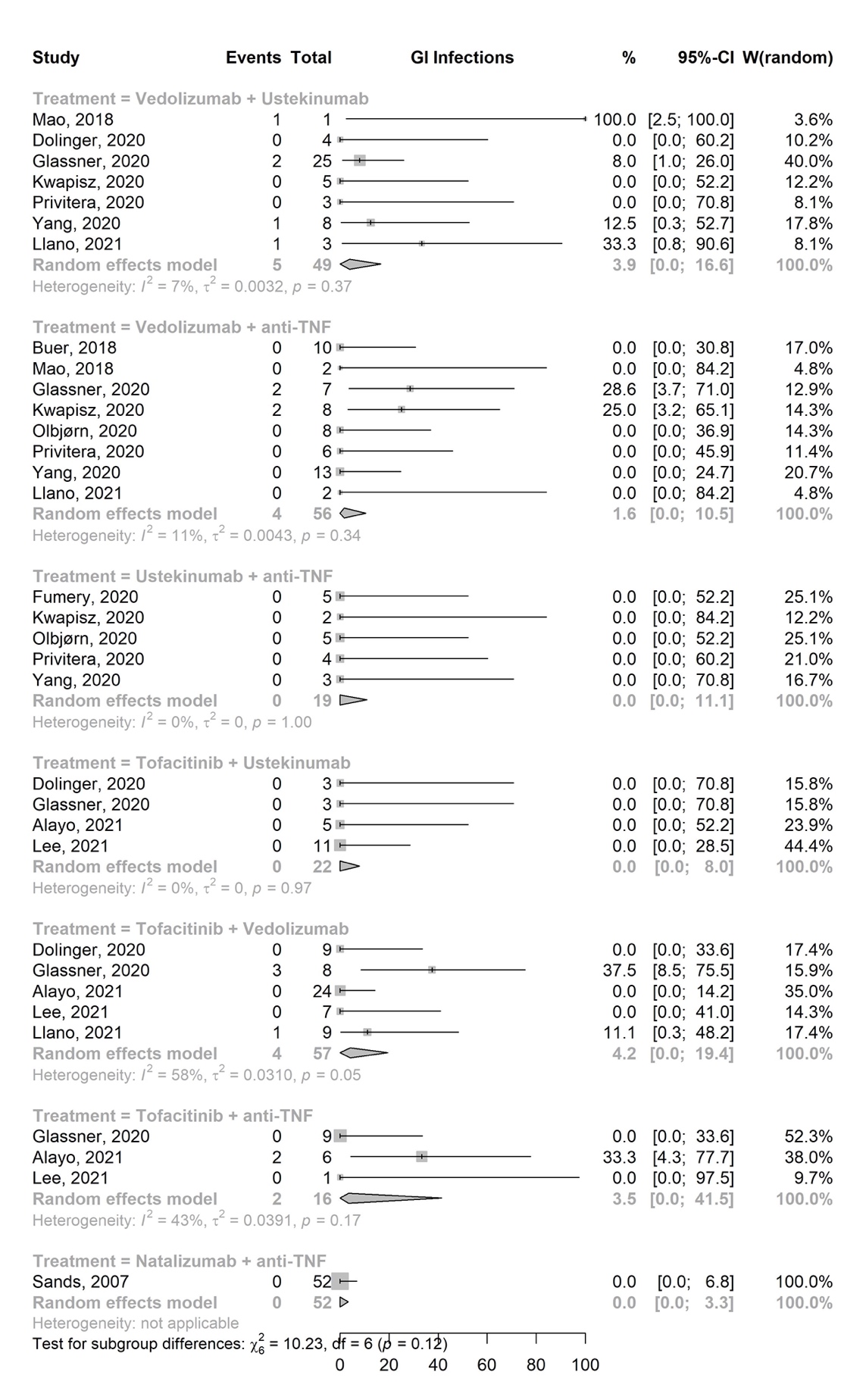


CI, Confidence interval; TNF, Tumour necrosis factor; W, Weights.

**Supplementary Figure 4: Forest plot of pooled clinical response rates for different combination therapies.**

**
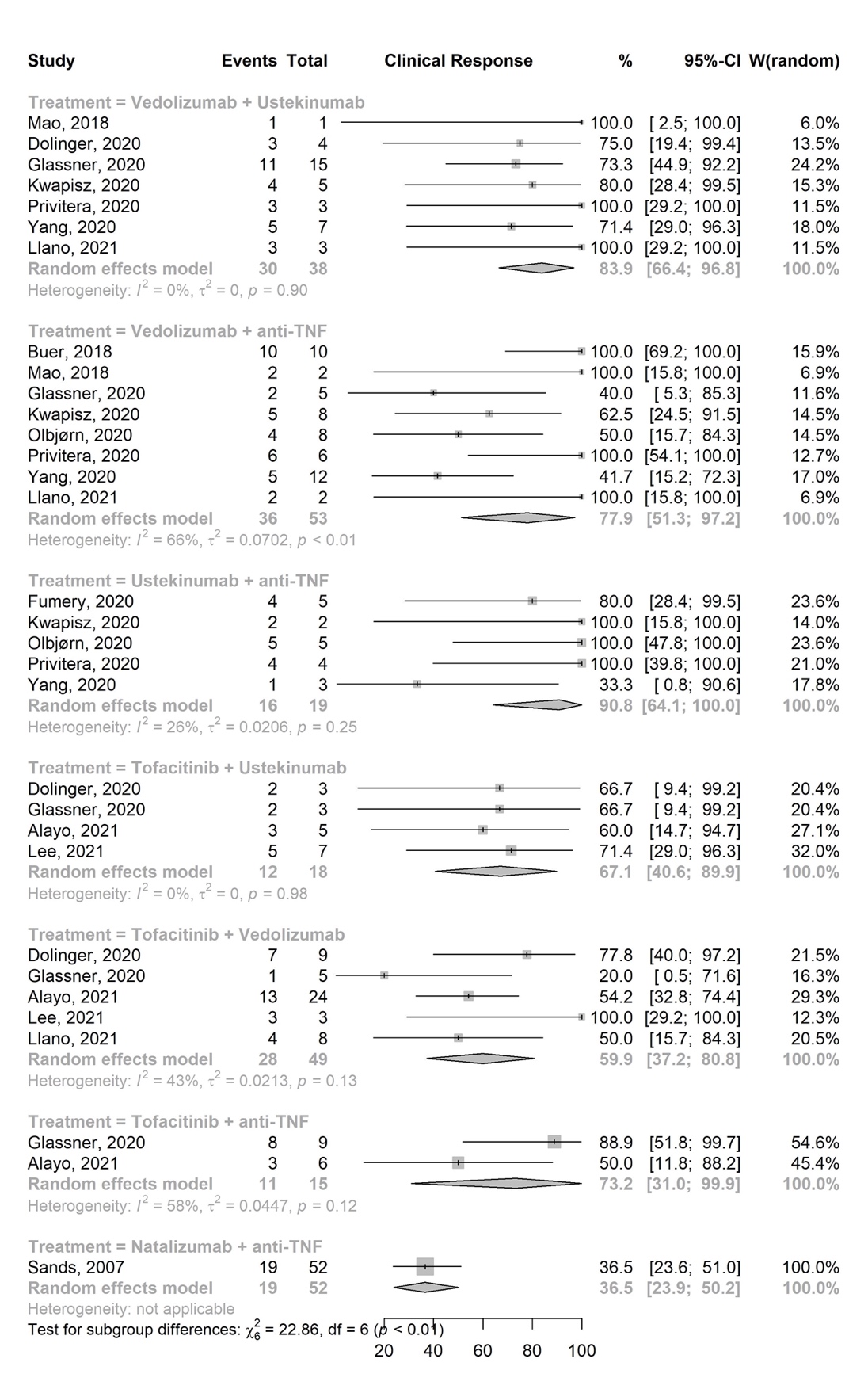
**

CI, Confidence interval; TNF, Tumour necrosis factor; W, Weights.

**Supplementary Figure 5: Clinical response data limited to patients with active luminal disease**


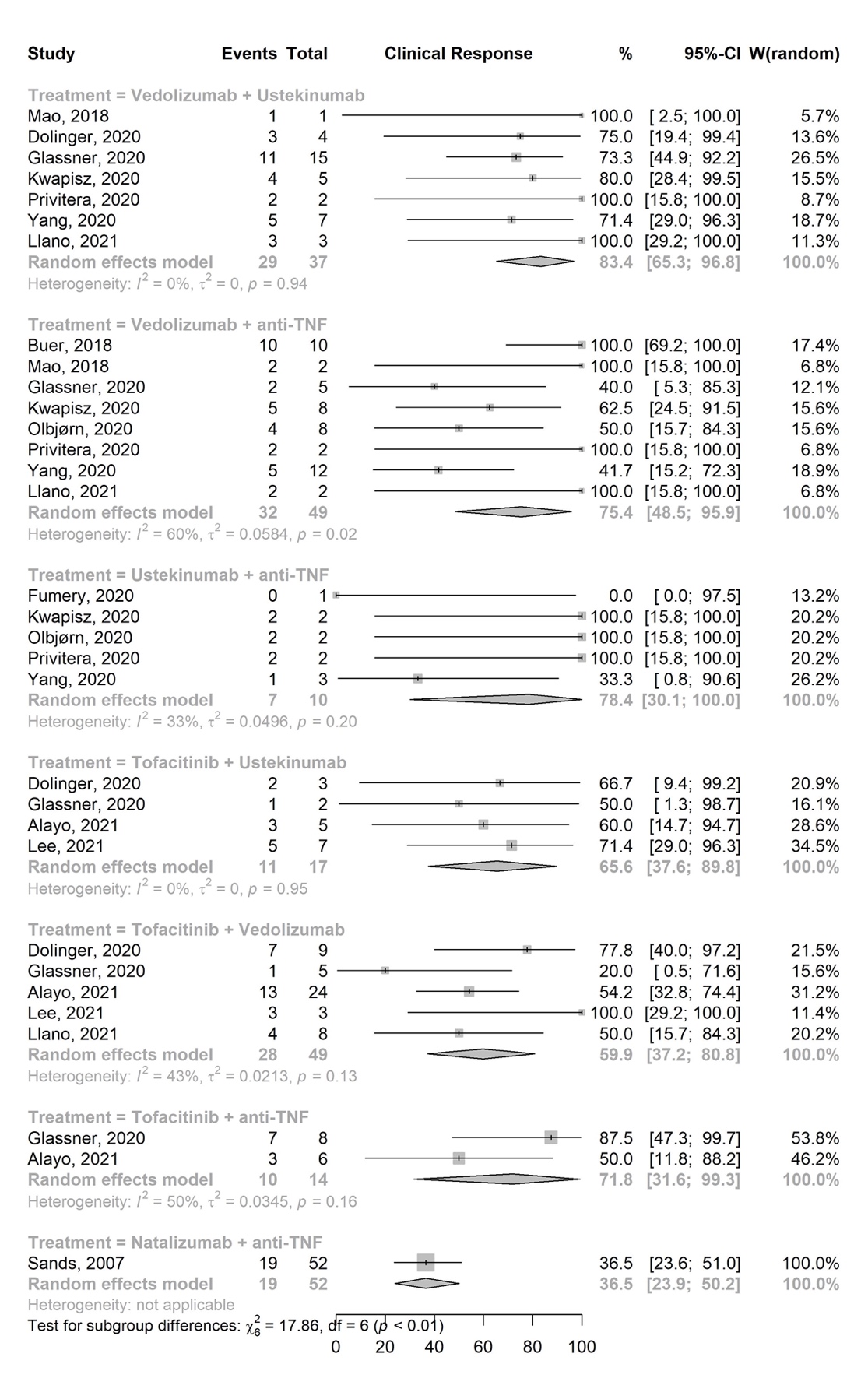


CI, Confidence interval; TNF, Tumour necrosis factor; W, Weights.

**Supplementary Figure 6: Clinical remission data limited to patients with active luminal disease**

**
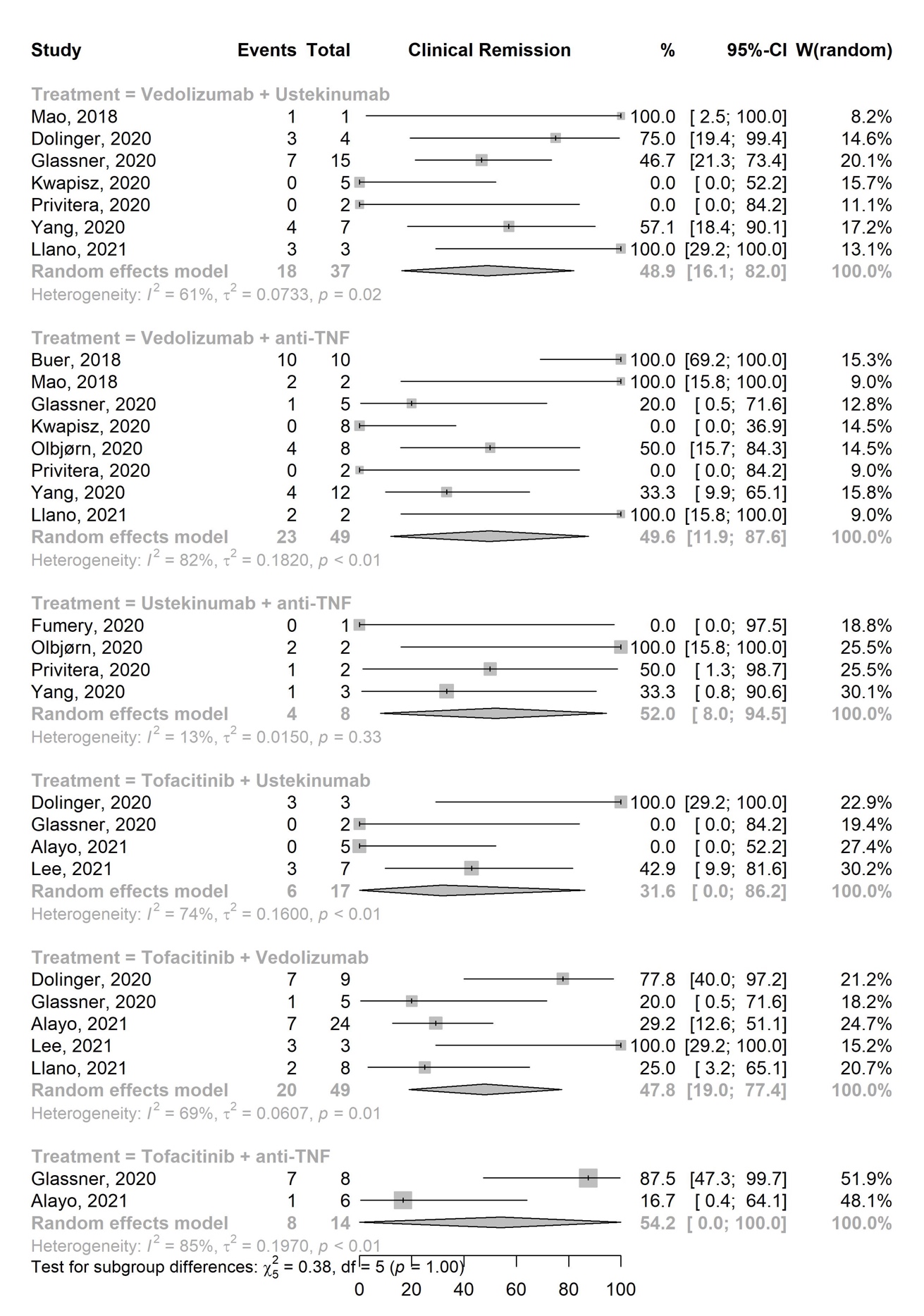
**

CI, Confidence interval; TNF, Tumour necrosis factor; W, Weights

**Supplementary Figure 7: Clinical response rate (generalized-linear mixed model)**


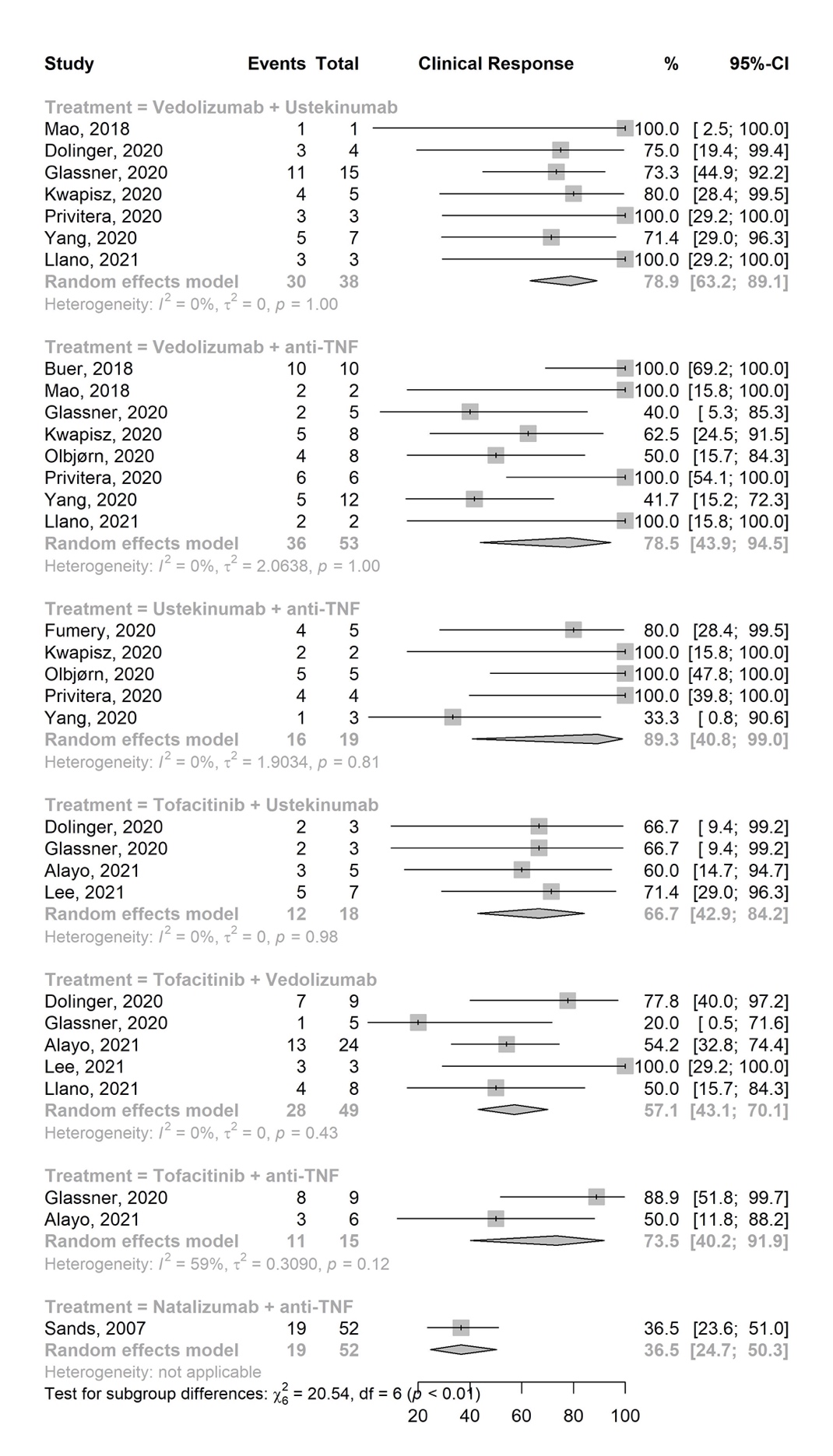


CI, Confidence interval; TNF, Tumour necrosis factor; W, Weights

**Supplementary Figure 8: Clinical remission rate (generalized-linear mixed model)**


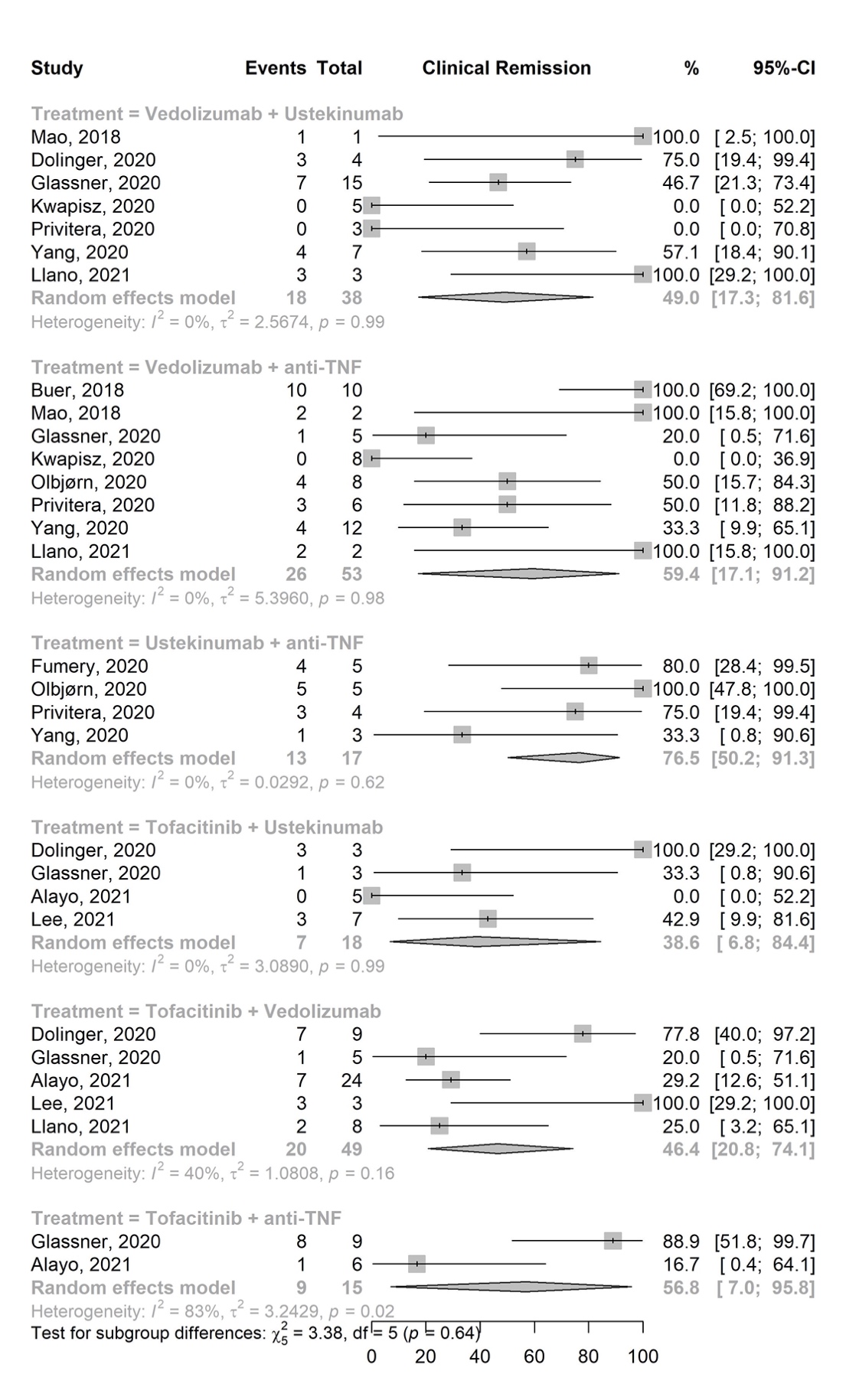


CI, Confidence interval; TNF, Tumour necrosis factor; W, Weights.

**Supplementary Figure 9: Forest plot of pooled endoscopic/radiologic response rates for different combination therapies.**

**
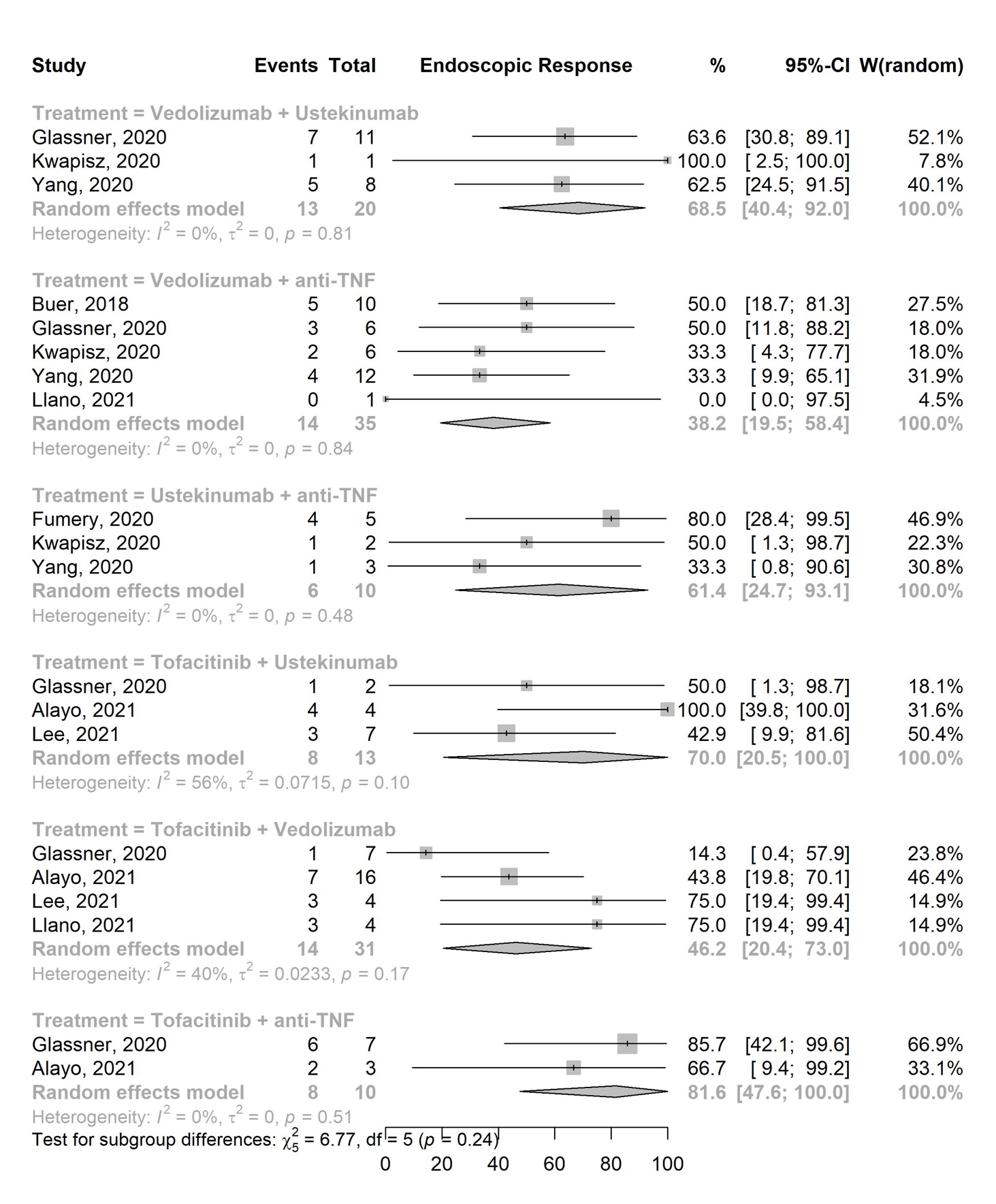
**

CI, Confidence interval; TNF, Tumour necrosis factor; W, Weights.

**Supplementary Figure 10: Forest plot of pooled endoscopic/radiologic remission rates for different combination therapies.**

**
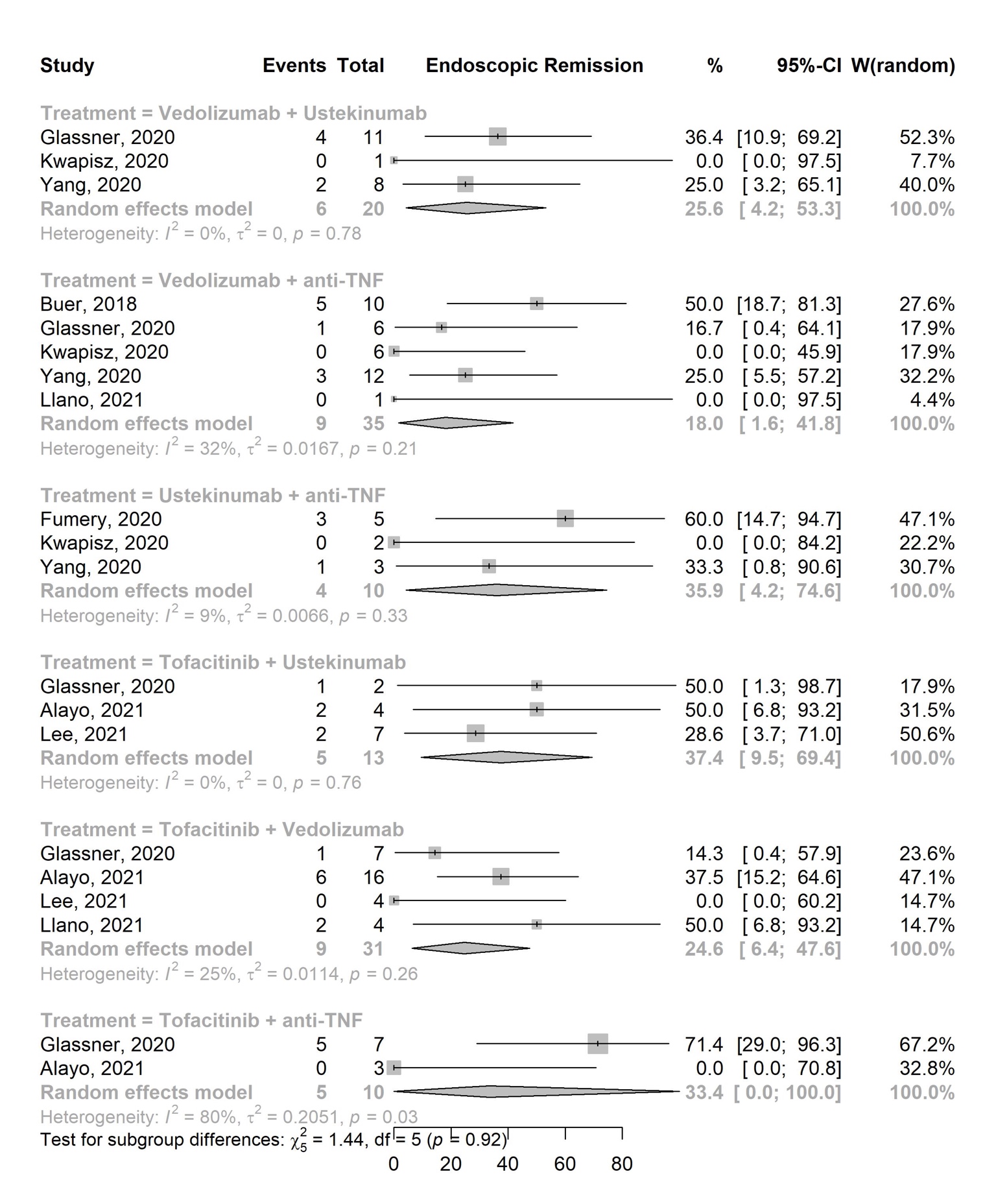
**

CI, Confidence interval; TNF, Tumour necrosis factor; W, Weights.

**Supplementary Figure 11: Endoscopic/radiological response imited to patients with active luminal disease**


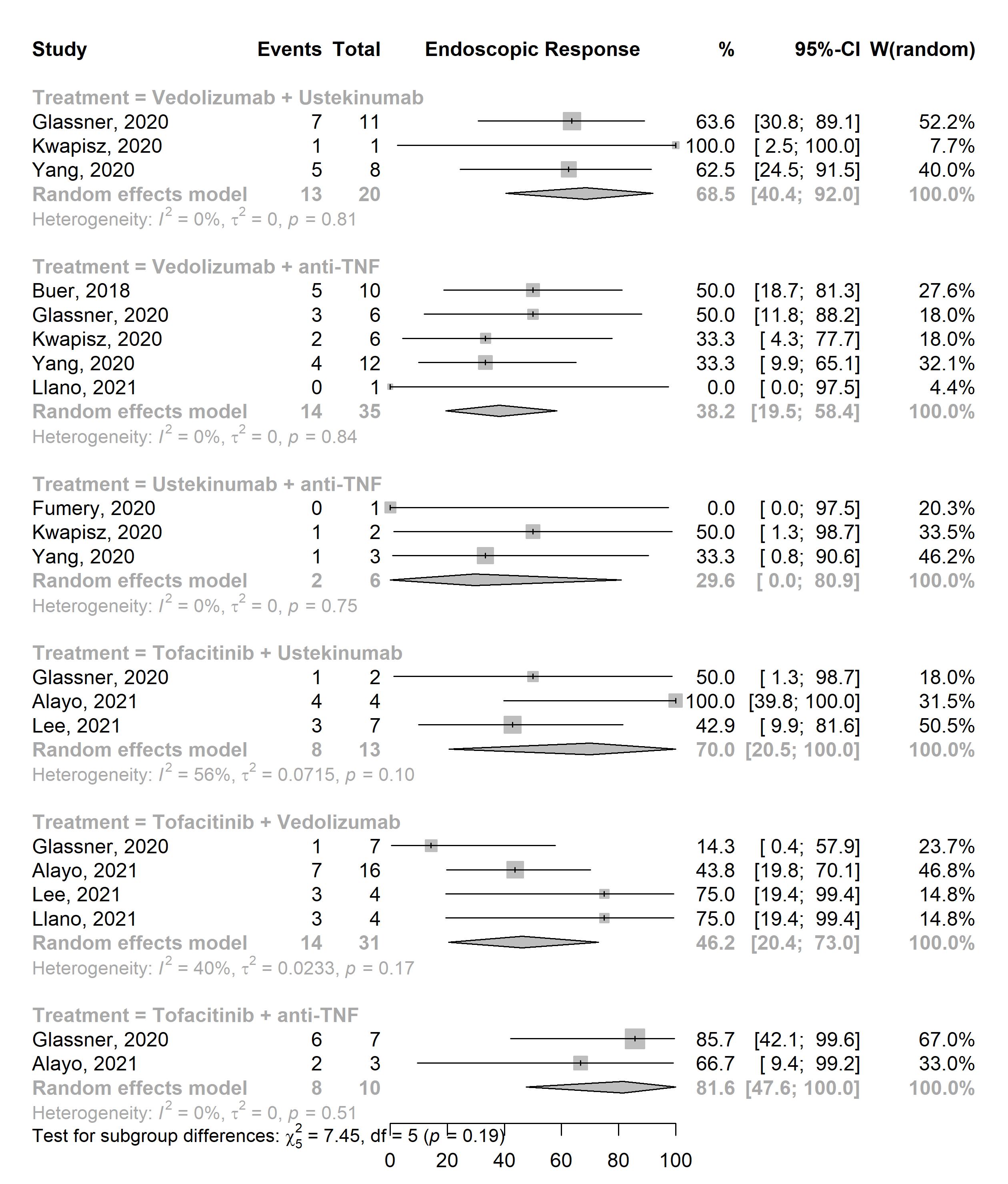


CI, Confidence interval; TNF, Tumour necrosis factor; W, Weights

**Supplementary Figure 12: Endoscopic/radiological remission data limited to patients with active luminal disease**


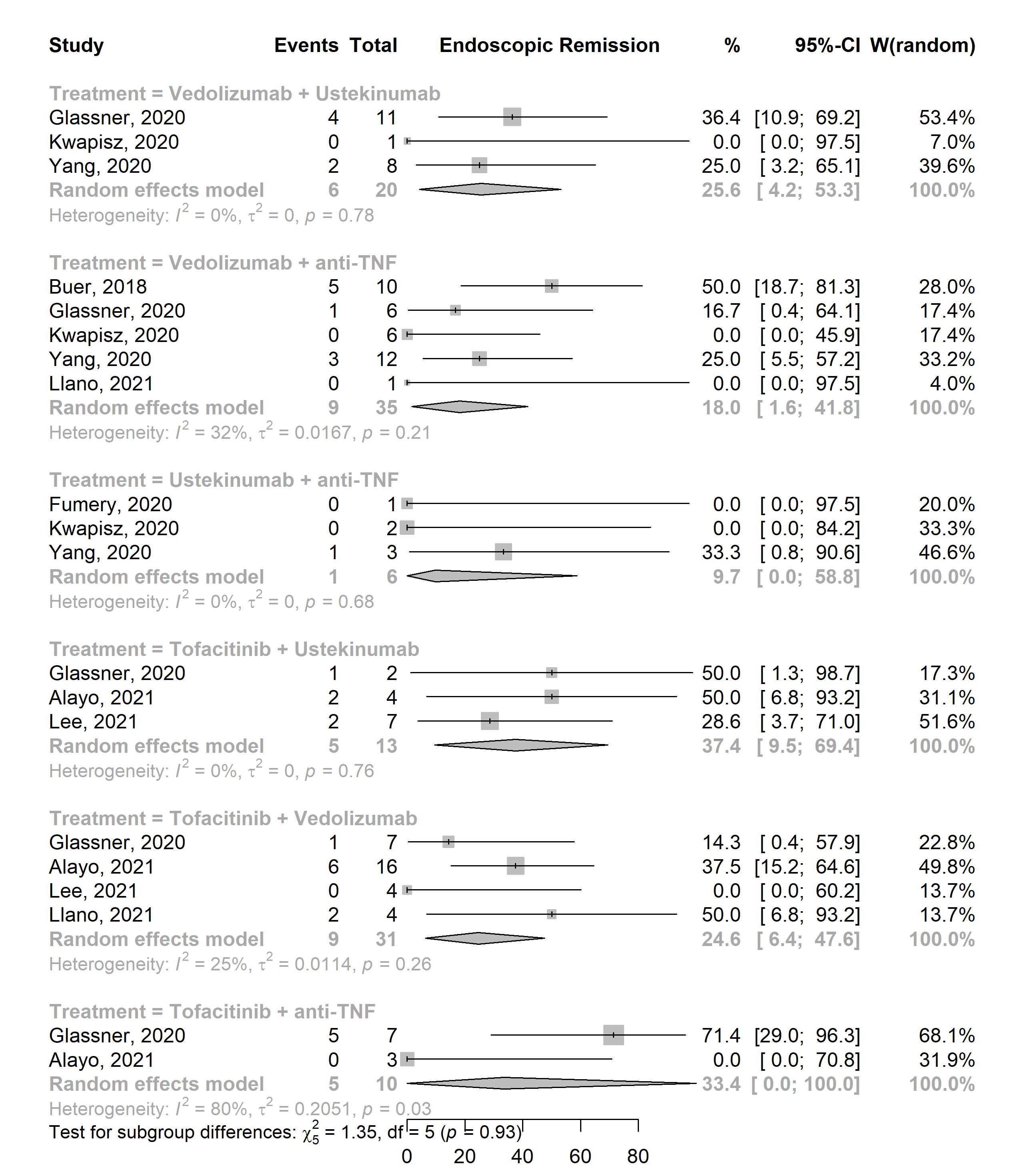


CI, Confidence interval; TNF, Tumour necrosis factor; W, Weights.

**Supplementary Figure 13: Endoscopic/radiological response rate (generalized-linear mixed model)**


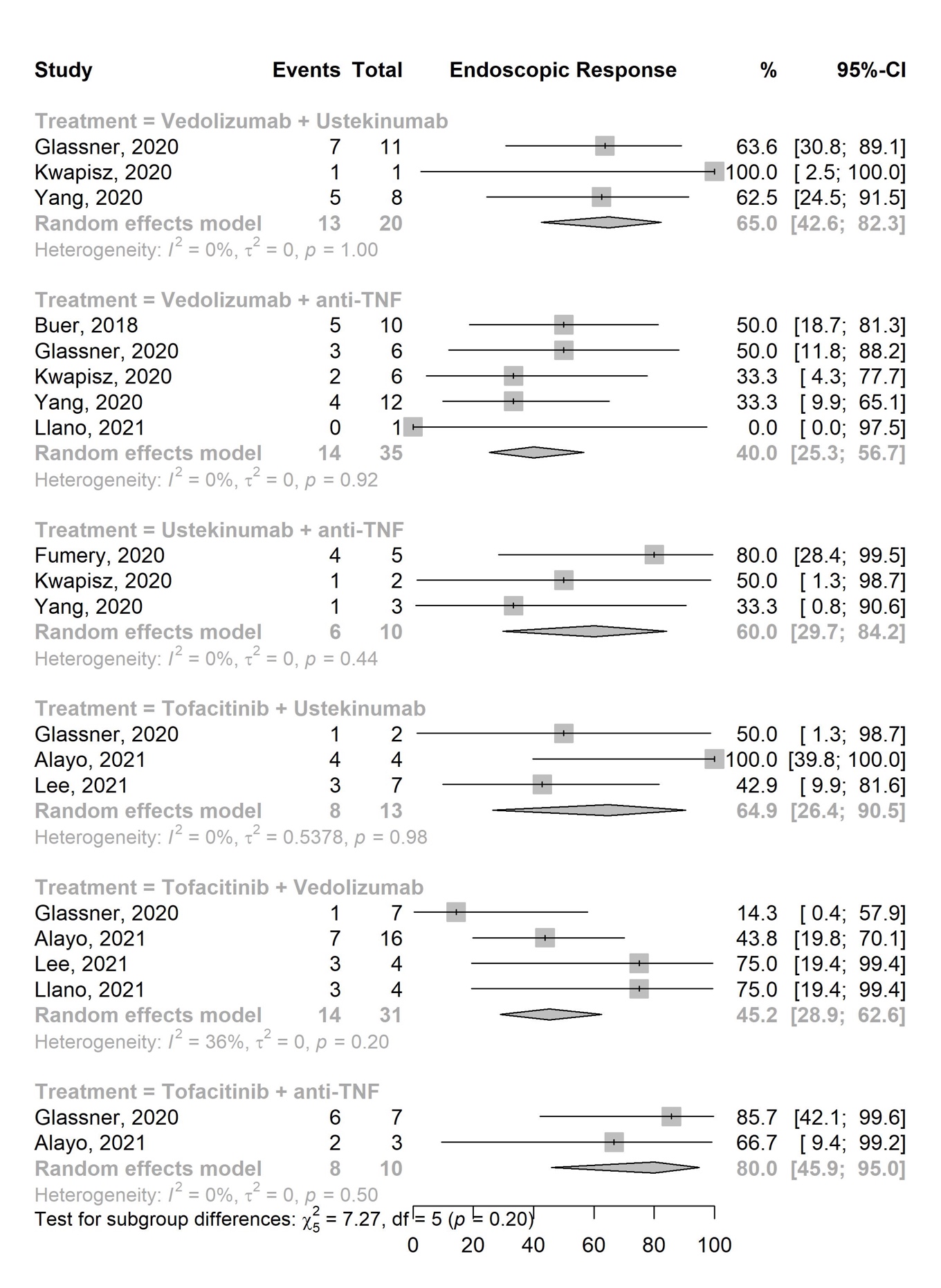


CI, Confidence interval; TNF, Tumour necrosis factor; W, Weights.

**Supplementary Figure 14: Endoscopic/radiological remission rate (generalized-linear mixed model)**


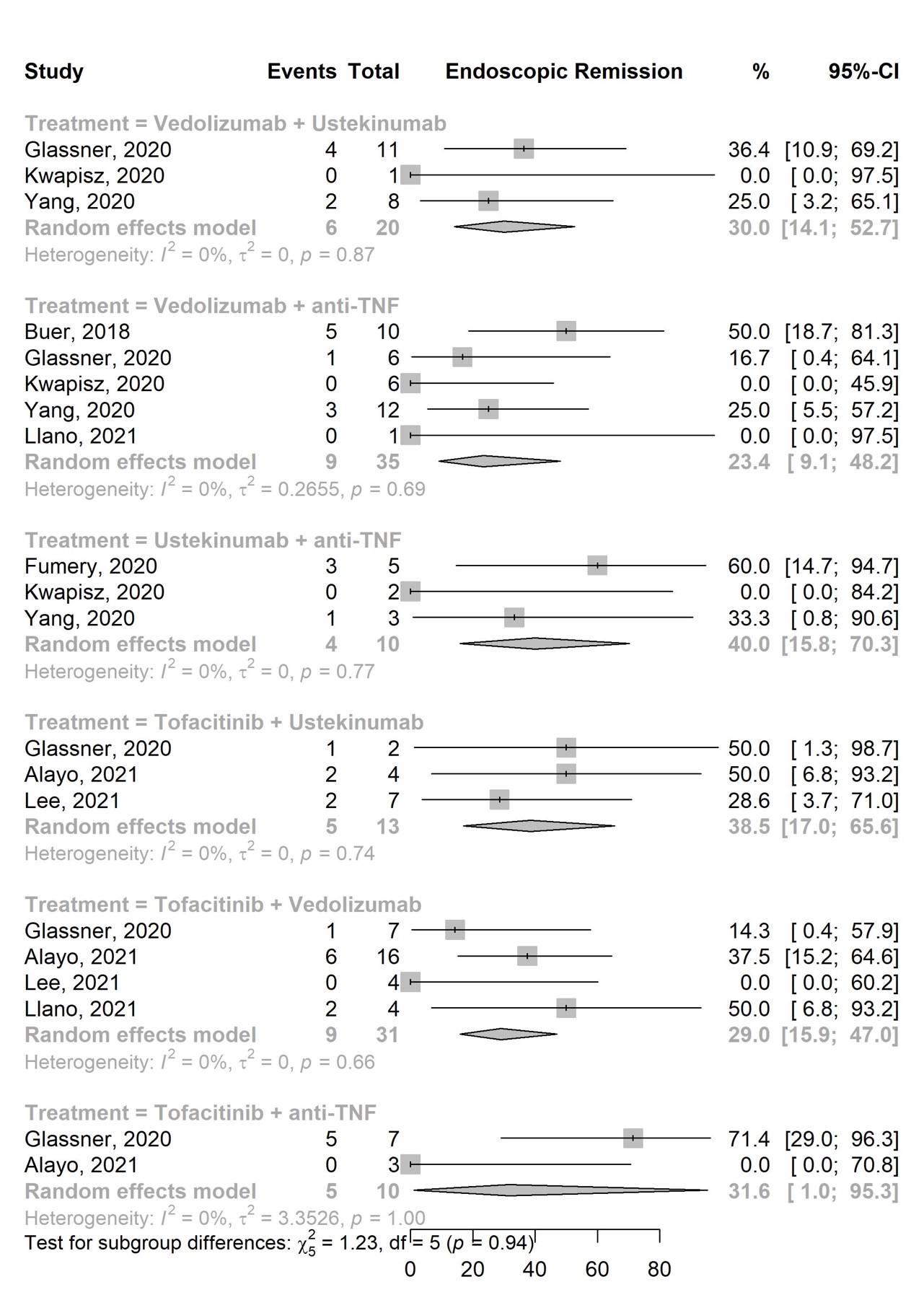


CI, Confidence interval; TNF, Tumour necrosis factor; W, Weights.

**REFERENCES**

1. Mao EJ, Lewin S, Terdiman JP, et al. Safety of dual biological therapy in Crohn's disease: a case series of vedolizumab in combination with other biologics. BMJ Open Gastroenterol. 2018;5:e000243.
2. Dolinger MT, Spencer EA, Lai J, et al. Dual Biologic and Small Molecule Therapy for the Treatment of Refractory Pediatric Inflammatory Bowel Disease. Inflamm Bowel Dis. 2020. https://doi.org/10.1093/ibd/izaa277 Published Oct 30, 2020
3. Glassner K, Oglat A, Duran A, et al. The use of combination biological or small molecule therapy in inflammatory bowel disease: A retrospective cohort study. *J Dig Dis.* 2020;21:264-271.
4. Kwapisz L, Raffals LE, Bruining DH, et al. Combination Biologic Therapy in Inflammatory Bowel Disease: Experience from a Tertiary Care Center. *Clin Gastroenterol Hepatol.* 2020. https://doi:10.1016/j.cgh.2020.02.017. Published Feb 14, 2020.
5. Llano EM, Shrestha S, Burstein E, et al. Favorable Outcomes Combining Vedolizumab With Other Biologics or Tofacitinib for Treatment of Inflammatory Bowel Disease. *Crohn's & Colitis 360.* 2021;3.
6. Privitera G, Onali S, Pugliese D, et al. Dual Targeted Therapy: A Possible Option for the Management of Refractory Inflammatory Bowel Disease*. J Crohns Colitis.* 2020. https://doi:10.1093/ecco-jcc/jjaa149. Published Jul 17, 2020
7. Yang E, Panaccione N, Whitmire N, et al. Efficacy and safety of simultaneous treatment with two biologic medications in refractory Crohn's disease. *Aliment Pharmacol Ther.* 2020;51:1031-1038.
8. Buer LCT, Hoivik ML, Warren DJ, et al. Combining Anti-TNF-alpha and Vedolizumab in the Treatment of Inflammatory Bowel Disease: A Case Series. Inflamm Bowel Dis. 2018;24:997-1004.
9. Olbjorn C, Rove JB, Jahnsen J. Combination of Biological Agents in Moderate to Severe Pediatric Inflammatory Bowel Disease: A Case Series and Review of the Literature. *Paediatr Drugs.* 2020;22:409-416.
10. Fumery M, Yzet C, Brazier F. Letter: combination of biologics in inflammatory bowel diseases. Aliment Pharmacol Ther. 2020;52:566-567.
11. Alayo QA, Khatiwada A, Patel A, et al. Effectiveness and Safety of Combining Tofacitinib with a Biologic in Patients with Refractory Inflammatory Bowel Diseases. *Inflamm Bowel Dis*. 2021 https://doi: 10.1093/ibd/izab112. Published May 25, 2021*.*
12. Lee SD, Singla A, Harper J, et al. Safety and Efficacy of Tofacitinib in Combination with Biologic Therapy for Refractory Crohn's Disease. *Inflamm Bowel Dis.* 2021. https://doi: 10.1093/ibd/izab176. Published Aug 4, 2021.
13. Sands BE, Kozarek R, Spainhour J, et al. Safety and tolerability of concurrent natalizumab treatment for patients with Crohn's disease not in remission while receiving infliximab. *Inflamm Bowel Dis.* 2007;13:2-11.
14. Moga C, Guo B, Schopflocher D, et al. Development of a Quality Appraisal Tool for Case Series Studies Using a Modified Delphi Technique. Institute of Health Economics. 2012.
